# Visible Light-Mediated Synthesis of Chalcogen-Decorated 2,3-Dihydrobenzofurans in the Absence of Photocatalyst and Oxidants

**DOI:** 10.1021/acsomega.5c12021

**Published:** 2026-03-04

**Authors:** Guilherme Araújo, Mateus W. Rambo, Sabrina S. Ferreira, Thiago Anjos, Ricardo F. Schumacher, Gelson Perin, Eder J. Lenardão, Filipe Penteado

**Affiliations:** † Centro de Ciências Químicas, Farmacêuticas e de Alimentos (CCQFA), 37902 Universidade Federal de Pelotas (UFPel), P.O. Box 354, 96010-900 Pelotas, RS, Brazil; ‡ Departamento de Química, Centro de Ciências Naturais e Exatas (CCNE), 28118 Universidade Federal de Santa Maria (UFSM), Av. Roraima no. 1000, 97105-340, Santa Maria, RS, Brazil

## Abstract

A sustainable visible light-promoted protocol for preparing selenium- and sulfur-decorated 2,3-dihydrobenzofurans from 2-allylphenols and diorganyl dichalcogenides is reported. The reaction proceeds under blue LEDs irradiation (Kessil PR160L, λ_max_ = 440 nm) under mild conditions, in the absence of photocatalysts or oxidants, in accordance with the green chemistry principles. Eighteen derivatives were obtained, covering electron-rich, electron-deficient, and polycyclic systems, with yields from 20% to 96%. The method is operationally simple, scalable, and tolerant to diverse aryl substituents, although the presence of electron-withdrawing groups reduces its efficiency. This approach offers an ecofriendly alternative to conventional oxidative or photocatalyst-mediated strategies to prepare the target molecules, opening new opportunities for advancing organoselenium chemistry and exploring its potential in organic synthesis.

## Graphical abstract

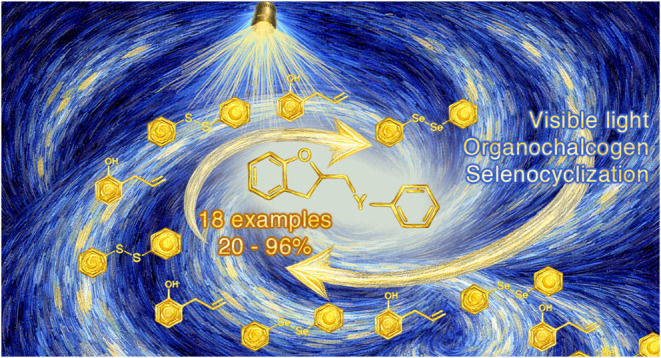

## Introduction

The imperative to develop environmentally sustainable processes, crystallized by the green chemistry principles (GCP) proposed by Anastas and Warner, has driven significant innovation in chemical research. In this multifaceted context, the energy supply in the chemical industry is a key subject, addressed by GCP #6 (design for energy efficiency), according to which conducting processes at room temperature is preferable, remarkably reducing the energy demand.^1^ Considering that many of the chemical processes established by the industry are energy-intensive, using alternative energy sources is an elegant approach to circumvent classical low energy efficiency heating apparatus. In this scenario, visible light-mediated reactions have emerged as a powerful and sustainable option, offering efficient pathways to organic transformations under mild reaction conditions by harnessing the energy of readily available light sources, like CFLs and LEDs.^2^

On the other hand, organoselenium compounds belong to a polyvalent class of compounds that have been employed in several important areas, including organic synthesis, biochemistry, materials science, and biotechnology.^3^ Most of the reactivity of these compounds is based on classical two-electron ionic chemistry by employing diorganyl diselenides as the standard selenium-precursor reagent. However, considering the stability of the Se–Se bond, the use of reducing or oxidant agents is mandatory to trigger the reactivity of these compounds, either by strategies employing Se-based nucleophiles (reductive cleavage of the Se–Se bond) or by using Se-based electrophiles (oxidative cleavage).^4^ In this context, light-mediated reactions have been opening new horizons in organoselenium chemistry, allowing the exploration of one-electron radical reactivity through the formation of Se-centered radical species. Regarding the ultraviolet–visible (UV–vis) absorption spectra of diaryl diselenide derivatives, the presence of an absorption tail in the UVA (λ_range_ ∼ 315–400 nm) and blue (λ_range_ ∼ 400–440 nm) allows to explore the homolytic cleavage of the Se–Se bond, even in the absence of reducing or oxidant agents, making this chemistry environmentally friendlier.^5^

2,3-Dihydrobenzofuran (2,3-DHB) is a class of naturally occurring heterocycles, widely found in many bioactive substances and presenting a plethora of biological applications. Furthermore, 2,3-DHB is found in the core structure of commercial drugs, used in the treatment of different disorders (Scheme 1, bioactive 2,3-DHB derivatives).^6^ Based on the impressive bioactive properties of 2,3-DHB, seminal studies have recently investigated the molecular hybridization improvements afforded by preparing selenium-decorated 2,3-DHB, some of which have presented potent inhibition of AChE and MAO-B enzymes, raising up an alternative class of compounds for the treatment of Alzheimer’s and Parkinson’s diseases (Scheme 1, selenium-decorated 2,3-DHB derivatives).^7^

**Scheme 1:**
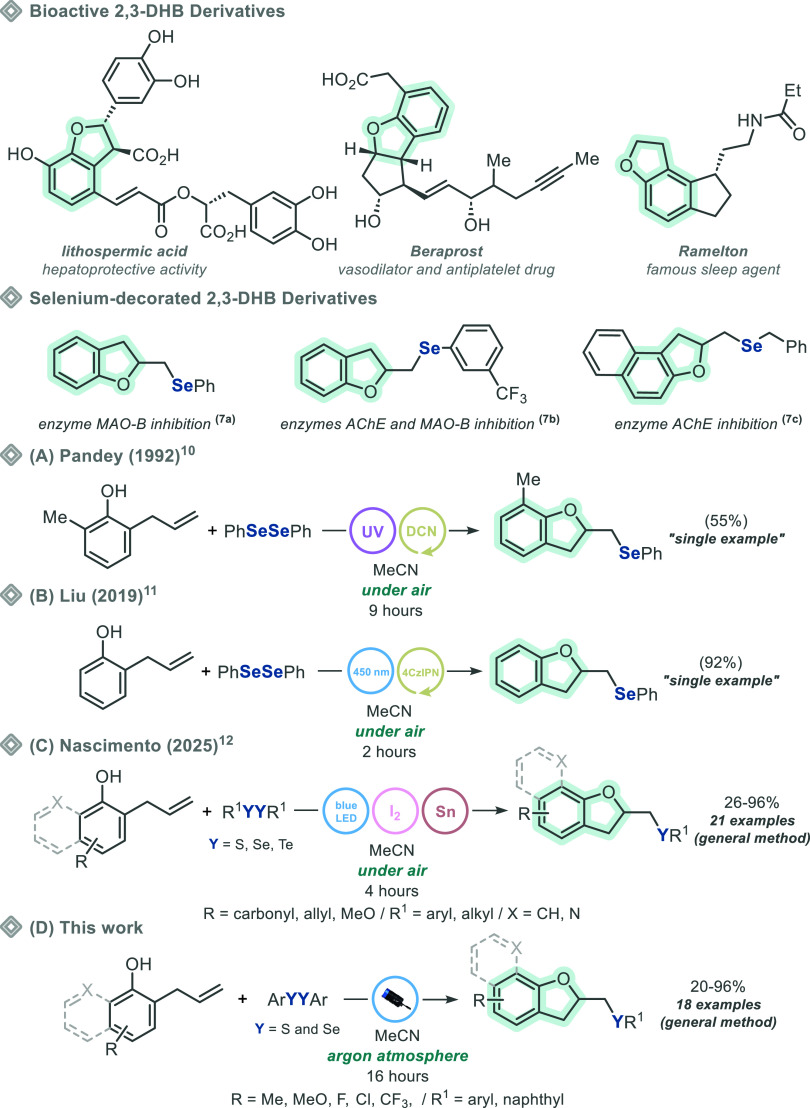
Bioactive 2,3-DHBs and Strategies to Prepare Selenium- and Sulfur-Decorated Derivatives

The synthesis of selenium-decorated 2,3-DHB has been extensively explored over the last years, by reacting diorganyl diselenides and 2-allylphenol derivatives under a diversity of approaches.^7b,8^ However, most of these strategies require harsh conditions, especially highly oxidative reaction media, which in general demand equivalent or overstoichiometric amounts of oxidant species. Parallelly, more attractive methods employing alternative energy sources have been emerging, especially under electrochemistry and microwave conditions.^7b,7c,9^

In this regard, some interesting approaches have been described under light irradiation conditions. In 1992, Pandey and Sekhar^10^ reported the synthesis of a single example of selenium-decorated 2,3-DHB derivatives by reacting diphenyl diselenide and 2-allylphenol under light irradiation. To achieve the desired products, the reaction was conducted in the presence of a photosensitizer (1,4-naphthalenedicarbonitrile, DCN) and required a high-power light source (450 W medium pressure lamb) emitting mostly UVA irradiation (λ_range_ ∼ 200–400 nm) (Scheme 1A). In 2019, Liu and co-workers^11^ reported the synthesis of one example of selenium-decorated 2,3-DHB, by a very similar strategy, however, in the presence of 4CzIPN (2 mol %) as photocatalyst, and under blue light irradiation (LEDs, λ_max_ = 450 nm). It is worth mentioning that even though this protocol is simple to operate, an important drawback is the complexity of the photocatalyst, which leads to economic inequivalence (Scheme 1B). More recently, while this work was in progress, a blue light-mediated approach to prepare selenium-decorated 2,3-DHB was described.^12^ The transformation is general and uses the same 2-allylphenol cyclization strategy. However, an important disadvantage is the need for using I_2_ (1 equiv) and SnCl_2_·2H_2_O (2 equiv) as reaction promoters, which contributes to the generation of huge amounts of waste at the end of the processes (Scheme 1C).

In this scenario, we report herein an ecofriendly approach to prepare chalcogen-decorated 2,3-DHB, by reacting diorganyl dichalcogenide and 2-allylphenol, in the presence of MeCN and argon atmosphere, the resulting mixture being irradiated exclusively with blue light (Kessil PR160L, λ_max_ = 440 nm), not requiring photocatalysts and/or oxidants, allowing the synthesis of 18 chalcogen-decorated 2,3-DHB derivatives in up to 96% yield (Scheme 1D).

## Results and Discussion

Aiming to optimize the reaction conditions, 2-allylphenol **1a** and diphenyl diselenide **2a** were selected as the standard substrates. Initially, an experiment was conducted to obtain 2-[(phenylselanyl)­methyl]-2,3-dihydrobenzofuran **3a** by stirring a mixture of **1a** (1.0 equiv, 0.3 mmol) and **2a** (1.0 equiv, 0.15 mmol) in MeCN (2.0 mL), under continuous irradiation with blue light (Kessil PR160L, λ_max_ = 440 nm). After 16 h under an air atmosphere (open flask), the desired product **3a** was obtained in 60% yield (Table 1, entry 1). Based on this result, we investigated the performance of other polar solvents, conducting experiments in the presence of EtOH and EtOAc. In both cases, the reaction efficiency decreased significantly, affording **3a** in only 20% and 35% yield, respectively (Table 1, entries 2 and 3). Next, the amount of diphenyl diselenide **2a** was adjusted from 0.15 to 0.20 and 0.30 mmol, to evaluate the performance of the reaction with an excess of **2a** (Table 1, entries 4 and 5). When 0.2 mmol of **2a** was used, product **3a** was obtained in 81% yield (Table 1, entry 4); however, using 0.3 mmol of **2a**, the yield of **3a** decreased to 62% (Table 1, entry 5).

**Table 1:**
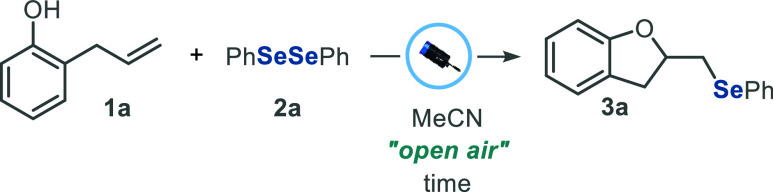
Reaction Optimization for the Synthesis of **3a** ^a^

#	**2a** (equiv)	time (h)	yield (%)^a^
1	1	16	60
2^b^	1	16	20
3^c^	1	16	35
4	1.3	16	81
5	2	16	62
6	1.3	24	45
7	1.3	8	34
8^d^	1.3	16	43
9^e^	1.3	16	51
10^f^	1.3	16	50
11^g^	1.3	16	79
**12** ^h^	1.3	**16**	**96**

a Into a reaction vial was added 2-allylphenol **1a** (1.0 equiv, 0.3 mmol), diphenyl diselenide **2a** (1 equiv = 0.15 mmol, 1.3 equiv = 0.2 mmol, 2.0 equiv = 0.3 mmol), and solvent (2.0 mL). The resulting mixture was stirred under blue light irradiation (Kessil PR160L, λ_max_ = 440 nm) for the tabled time. After the reaction completion, product **3a** was purified by column chromatography. The final internal reaction temperature was around 30–35 °C.

b EtOH was the solvent.

c EtOAc was the solvent.

d Used 1.0 mL of MeCN.

e Used 3.0 mL of MeCN.

f UVA light (Kessil PR160L, λ_max_ = 390 nm) was used.

g White light (LEDs 50 W) was used.

h Ar atmosphere.

The influence of reaction time was also investigated, with the transformation carried out for 24 and 8 h. In both cases, no improvement was observed, affording product **3a** in 45% and 34% yield, respectively (Table 1, entries 6 and 7). The effect of concentration was then examined, with two additional experiments performed using 1.0 and 3.0 mL of MeCN as solvent. No improvements were achieved, and product **3a** was obtained in 43% and 51% yield, respectively (Table 1, entries 8 and 9). The light source was also modified, replacing blue light with UVA (Kessil PR160L, λ_max_ = 390 nm) and white light (LEDs, 50 W) irradiation. Product **3a** was obtained in 50% and 79% yield, respectively, not surpassing the efficiency achieved with blue light (Table 1, entries 10 and 11). Finally, the transformation was carried out in the absence of oxygen by purging the reaction vial with argon. In this case, product **3a** was isolated in 96% yield, representing a remarkable improvement likely due to the suppression of parallel oxidation promoted by atmospheric O_2_ (Table 1, entry 12).

After establishing the best reaction conditions (Table 1, entry 12), we evaluated the efficiency of the protocol with a range of electron-rich and electron-deficient derivatives of substrates **1** and **2**. Initially, substituted diaryl diselenides **2b**–**f** were reacted with 2-allylphenol **1a**. Electron-donating groups (Ar = *p*-tolyl and *p*-anisole) afforded products **3b** and **3c** in 86% and 94% yield, respectively. In contrast, the presence of halogen atoms in the aromatic ring (Ar = *p*-fluorophenyl **2d** and *p*-chlorophenyl **2e**) significantly reduced the efficiency, affording products **3d** and **3e** in 51% and 45% yield, respectively. A similar decrease in reactivity was observed for the strong electron-withdrawing trifluoromethyl group (Ar = 3-(trifluoromethyl)­phenyl **2f**), which gave product **3f** in 58% yield (Table 2).

**Table 2:**
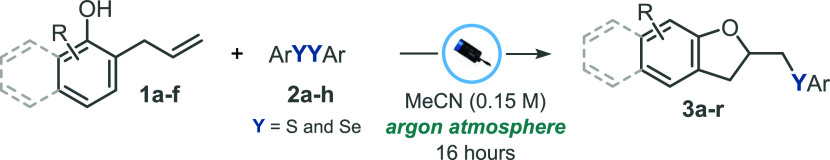

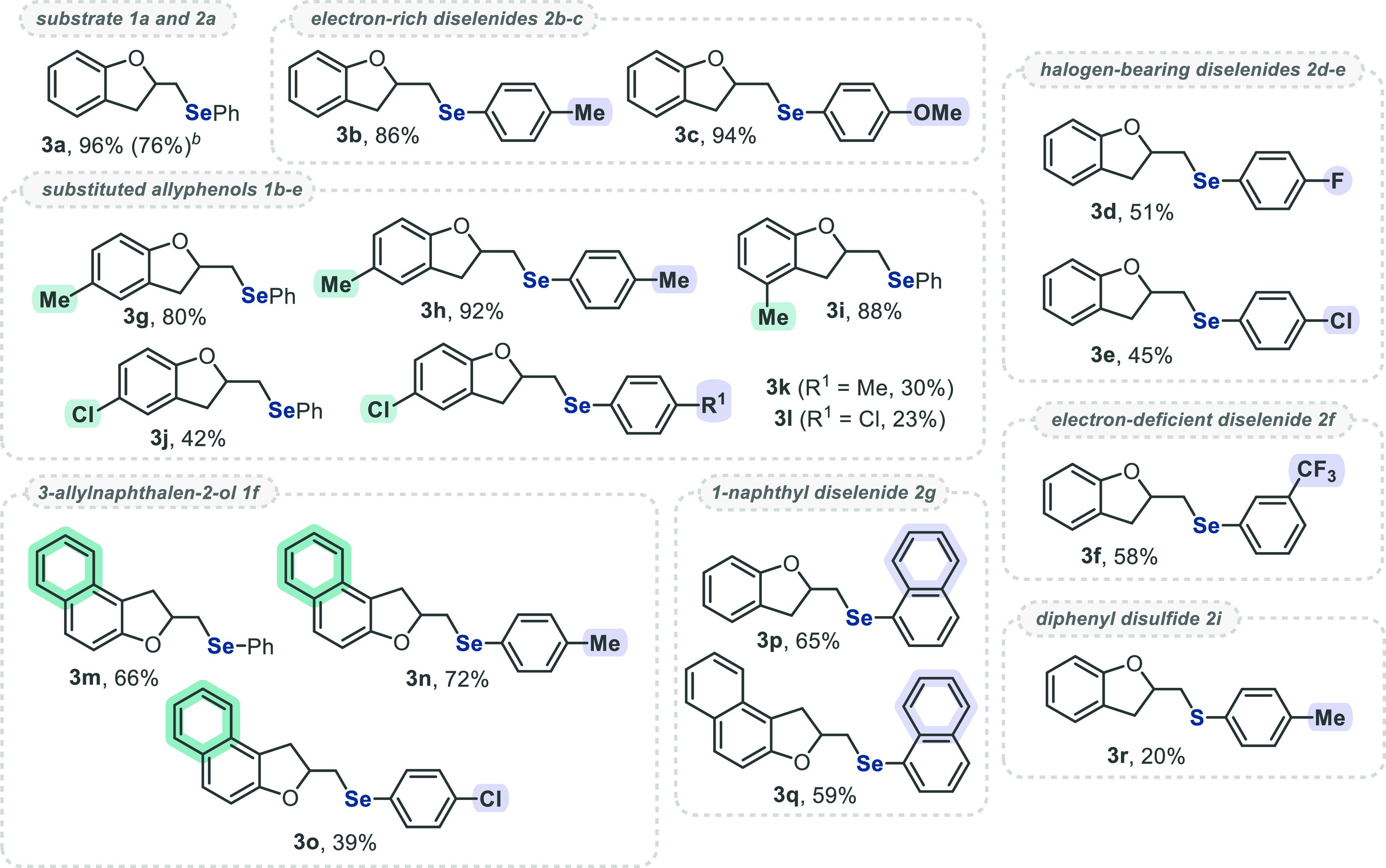
Reaction Scope Study for the Synthesis of Products **3a**-**r** ^a^,^b^

a Into a reaction vial was added the 2-allylphenol derivative **1** (1.0 equiv, 0.3 mmol), diaryl diselenide **2** (1.3 equiv, 0.2 mmol), and MeCN (2.0 mL). The resulting mixture was stirred under blue light irradiation (Kessil PR160L, λ_max_ = 440 nm) for 16 h. After the reaction completion, product **3** was purified by column chromatography. The final internal reaction temperature was around 30–35 °C.

b Reaction was performed using 3.0 mmol of 2-allylphenol **1a** and 2.0 mmol of diphenyl diselenide **2a**.

Subsequently, methyl-substituted allylphenols (R = 4-Me **1b** and 3-Me **1c**) were employed as substrates in the reaction with diaryl diselenides **2a** and **2b**. These combinations afforded products **3g**, **3h**, and **3i** in 80%, 92%, and 88% yield, respectively. Conversely, 2-allyl-chlorophenol **1d** reacted with **2a**, **2b**, and **2e** to produce **3j**, **3k**, and **3l** in 42%, 30%, and 23% yield, respectively. These results indicate that the presence of electron-withdrawing substituents such as fluorine, chlorine, and trifluoromethyl reduced the reactivity, while electron-donor substituents performed efficiently (Table 2).

Following this trend, 3-allylnaphthalen-2-ol **1e** was reacted with diselenides **2a**, **2b**, and **2e**, affording **3m**, **3n**, and **3o** in 66%, 72%, and 39% yield, respectively. Additionally, 1-naphthyl diselenide **2g** reacted smoothly with **1a** and **1e**, producing **3p** and **3q** in 65% and 59% yield, respectively. Unfortunately, dialkyldiselenides, including dibenzyldiselenide, were not suitable substrates under the optimized reaction conditions, as no formation of desired 2,3-dihydrobenzofuran products **3** was observed. Finally, diphenyl disulfide **2h** was submitted to the optimized conditions, providing product **3r** in only 20% yield. The lower reactivity of disulfide compared to diselenide can be attributed both to the stronger S–S bond and the absorption spectra of diphenyl disulfide, mostly in the UV region.^13^ These results demonstrate the broad applicability of the method to a range of substrates with diverse electronic properties (Table 2).

To gain insights into the mechanism of this cyclization reaction, a series of control experiments was performed under modified conditions. When the reaction was carried out in the absence of light (dark conditions), no product formation was observed, and substrates **1a** and **2a** were fully recovered (Scheme 2, experiment A). By performing the reaction under thermal conditions (refluxing MeCN), compound **3a** was obtained in only 19% yield, indicating that the transformation is mainly driven by photon absorption (Scheme 2, experiment B). This result rules out the possibility that thermal energy from the light source could drive the transformation, as the final internal reaction temperature remains about 30–35 °C. The addition of radical scavengers (4 equiv of TEMPO or 2 equiv of DPE) completely suppressed the reaction. Although no TEMPO–SePh adduct has been detected, GC–MS analysis confirmed the formation of the DPE–SePh adduct (*m*/*z* = 336) in approximately 85% conversion from diselenide **2a** (Scheme 2, experiments C and D. See Figure S1, in the Supporting Information file). This observation supports the involvement of a radical pathway and strongly suggests the possible formation of Se-centered radical species during the reaction course. This hypothesis is reinforced by the marked loss of efficiency observed when electron-deficient diselenides **2d**, **2e**, and **2f** were used as substrates, a factor that would destabilize the formed Se-centered radical. Complementary UV–vis analysis ruled out the formation of an EDA-complex between the reactants. Instead, diphenyl diselenide **2a** displayed a pronounced absorption tail (λ_tail_ > 400 nm), which enables its direct excitation by blue light, triggering the homolytic cleavage of the Se–Se bond (Figure S3, SI file).

**Scheme 2:**
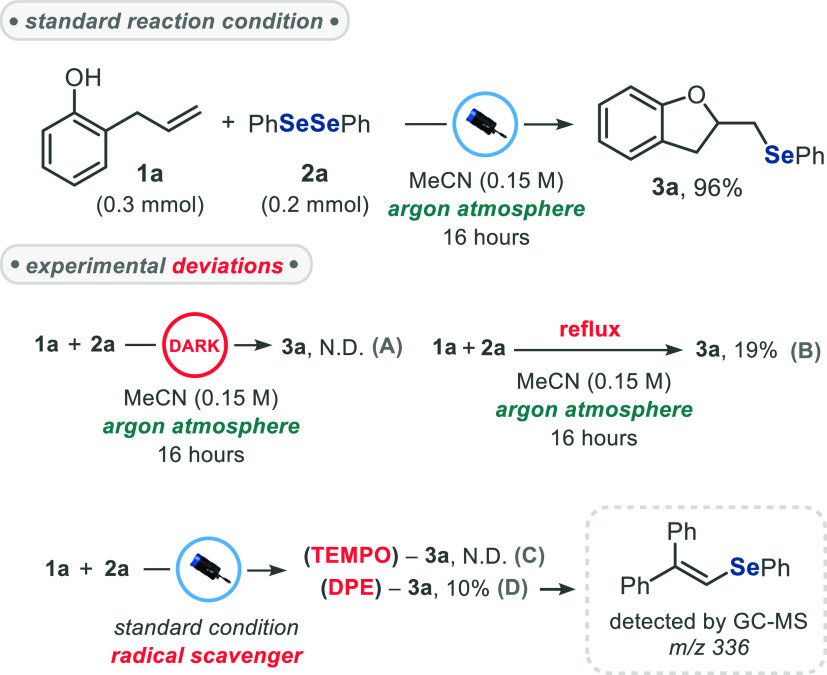
Control Experiments for Mechanistic insights

Based on these experimental observations and literature reports,^14^ a plausible mechanism was proposed. Initially, the homolytic cleavage of the Se–Se bond in **2a** is induced by the blue light, generating the selenium-centered radical **I**.^14a^ This radical adds to the C=C double bond of 2-allylphenol **1a**, forming alkyl radical intermediate **II**, which is then trapped by **2a** to yield vicinal diselenide intermediate **III**. In the sequence, an intramolecular S_N_2 reaction occurs, forming seleniranium-like species **IV**. Subsequently, the intermediate **IV** undergoes a *5-exo-tet* cyclization (favored according to Baldwin’s rules) to give the intermediate **V**, which is finally converted into the desired product **3a** (Scheme 3).

**Scheme 3:**
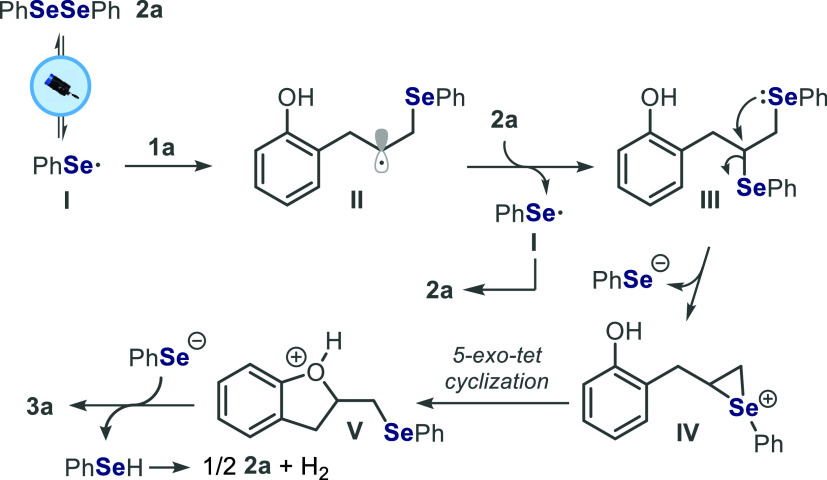
Plausible Reaction Mechanism

## Conclusions

In conclusion, we have demonstrated an efficient blue light-driven approach for the synthesis of selenium- and sulfur-decorated 2,3-dihydrobenzofurans **3** from 2-allylphenols **1** and diorganyl dichalcogenides **2**. This protocol accommodates a wide variety of reaction partners, delivering 18 distinct derivatives in yields of up to 96%. By dispensing with photocatalysts, oxidants, and other additives, the procedure minimizes waste generation and streamlines the overall operation. The transformation proceeds at room temperature in MeCN under visible-light irradiation, avoiding any additional thermal input. The combination of high efficiency, broad substrate scope, and operational simplicity underscores the method’s alignment with the green chemistry principles. Moreover, experimental evidence points to selenium-centered radical intermediates, suggesting promising opportunities for expanding radical methodologies in organoselenium chemistry and the development of new light-mediated chalcogenation strategies.

## Experimental Section

### General Information

The reactions were monitored by TLC carried out on Merk silica gel (60 F_254_) by using UV light as a visualization agent, and the mixture between 5% of vanillin in 10% of H_2_SO_4_ under heating conditions as a developing agent. Merck silica gel (particle size 0.040–0.063 mm) was used for flash chromatography. Hydrogen nuclear magnetic resonance spectra (^1^H NMR) were obtained on Bruker Avance III HD 400 MHz employing a direct broadband probe at 400 MHz. The spectra were recorded in CDCl_3_ solutions. The chemical shifts are reported in parts per million, referenced to tetramethyl silane (TMS) (0.00 ppm) as the internal reference. Coupling constants (*J*) are reported in Hertz. Abbreviations to denote the multiplicity of a particular signal are s (singlet), d (doublet), dd (doublet of doublet), t (triplet), q (quartet), quint (quintet), sext (sextet), td (triplet of doublet), and m (multiplet). Carbon-13 nuclear magnetic resonance spectra (^13^C NMR) were obtained on Bruker Avance III HD 400 MHz by employing a direct broadband probe at 100 MHz. The chemical shifts are reported in parts per million, referenced to the solvent peak of CDCl_3_ (δ 77.16 ppm) or DMSO-*d*_6_ (δ 39.52 ppm). High-resolution mass spectra were recorded using a mass spectrometer equipped with electrospray ionization (ESI), with samples injected by flow injection analysis (FIA) into an LCMS Q-TOF (model 9050, Shimadzu, Kyoto, Japan) controlled by OPLC Shimadzu Nexera Series (Shimadzu, Kyoto, Japan). Melting point (mp) values were measured in a Marte PFD III instrument. UV–vis absorption spectroscopy was recorded using a Shimadzu UV-2600 spectrophotometer (data interval = 1.0 nm and slit = 1.0 mm). Solvents and auxiliaries were purified by standard procedures (distillation and drying).

### General Procedure for the Synthesis of the 2-Allylphenol Derivatives **1a**–**f**

In a round-bottom flask (100 mL), phenol (0.94 g, 10 mmol, 1.0 equiv) was dissolved in anhydrous acetone (15 mL). Potassium carbonate (2.07 g, 15 mmol, 1.5 equiv) and allyl bromide (0.95 mL, 11 mmol, 1.1 equiv) were then added, and the resulting mixture was heated at 65 °C under stirring overnight. After cooling to room temperature, the reaction mixture was filtered and the solvent was removed under reduced pressure. The crude residue was dissolved in CH_2_Cl_2_ (5 mL) and sequentially washed with 1 M NaOH (5 mL), water (5 mL), and brine (5 mL). The organic layer was dried over anhydrous Na_2_SO_4_, filtered, and concentrated in vacuo. The crude product was purified by column chromatography on silica gel, employing a gradient of hexane/EtOAc (100:0 → 98:2) as the eluent.^15^

### General Procedure for the Synthesis of the Diorganyl Diselenides **2a**–**i**

In a flame-dried round-bottom flask under an inert atmosphere (argon), magnesium turnings (56.5 mmol) were suspended in freshly distilled anhydrous THF (30 mL), and a catalytic amount of I_2_ (1 to 2 small crystals) was added to initiate the reaction. A solution of the corresponding halobenzene (54 mmol) in freshly distilled anhydrous THF (30 mL) was then added dropwise under stirring. The reaction mixture was stirred until the complete formation of the Grignard reagent (1 h), as indicated by the full consumption of the magnesium turnings. Elemental selenium (54 mmol) was then added portionwise, and the resulting mixture was stirred at room temperature for 1 h. Subsequently, the reaction mixture was exposed to air, and a saturated aqueous NH_4_Cl solution (15 mL) was added. The mixture was stirred under open-air conditions overnight. The reaction mixture was extracted with EtOAc, and the combined organic layers were washed with water, dried over anhydrous MgSO_4_, filtered, and concentrated under reduced pressure. The product was crystallized from hexanes.^16^

### General Procedure for the Synthesis of the Derivatives **3a**–**r**

In a test tube, 2-allylphenol derivatives **1a**–**f** (0.3 mmol), diorganyl diselenides **2a**–**i** (0.2 mmol), and MeCN (2.0 mL) were mixed up. The vial was then purged with argon, and the resulting mixture was vigorously stirred under continuous blue light irradiation (Kessil PR160L, λ_max_ = 440 nm) for 16 h. During the process, a constant flow of argon was maintained by using a balloon. After this period, the LED lamp was switched off, and MeCN was removed using a rotary evaporator, followed by vacuum drying. The crude product was purified by column chromatography on silica gel, employing hexane/ethyl acetate (98:2) as the eluent. Yields, figures of NMR spectra, and characterization data of prepared compounds are available in the Supporting Information file (pages S6–S40).

## Supplementary Material



## Notes

Scale-up procedure for the synthesis of product **3a**; spectral data (^1^H, ^13^C, ^77^Se NMR) for compounds **3a–r** (Figures S4–S53); HRMS data for compounds **3h**, **3i**, **3k**, **3l**, **3n**, and **3p**; detailed experimental procedure and GC-MS spectrum of the radical scavenger experiment with DPE (Figure S1); visible light photoreaction setup (Figure S2); UV–vis analysis of compounds **1a** and **2a** (Figure S3) (PDF)
