# Supplementary material for: Visible Light-Mediated Synthesis of Chalcogen-Decorated 2,3-Dihydrobenzofurans in the Absence of Photocatalyst and Oxidants
Source: ACS Omega. 2026 Mar 4;11(10):16406–13. doi: 10.1021/acsomega.5c12021 (PMC13000581; doi:10.1021/acsomega.5c12021)
Supplement: Supplementary file 1 [file ao5c12021_si_001.pdf]

Visible Light-Mediated Synthesis of Chalcogen-Decorated 2,3-Dihydrobenzofurans in the Absence of Photocatalyst and Oxidants

Guilherme Araújo,[a] Mateus W. Rambo,[b] Sabrina S. Ferreira,[a] Thiago Anjos,[b] Ricardo F. Schumacher,[b] Gelson Perin,[a] Eder J. Lenardão,[a,*] and Filipe Penteado[b,*]

[a] Centro de Ciências Químicas, Farmacêuticas e de Alimentos (CCQFA), Universidade Federal de Pelotas (UFPel), P.O. Box 354, 96010-900, Pelotas, RS, Brazil.

[b] Departamento de Química, Centro de Ciências Naturais e Exatas (CCNE), Universidade Federal de Santa Maria (UFSM), Av. Roraima, n° 1000, 97105-340, Santa Maria, RS, Brazil.

Table of Contents

1 Scale-up procedure for the synthesis of product 3a..S3

2. Radical scavenger experiments………………………………………….………….S3

3. Visible-light photoreaction setup…………………………….…………………..…..S3

4. Uv-Vis analysis of compounds 1a and 2a………………………..…………………S4

5. Spectral data for the compounds 3a–3r……………………………………………..S6

6. References…………………………………………………………………………...S13

7. Selected Spectra…………………………………………………….………………S14

1. Scale-up procedure for the synthesis of product 3a

In a test tube, 2-allylphenol **1a** (3 mmol), diphenyl diselenide **2a** (2 mmol), and MeCN (20.0 mL) were mixed up. The vial was then purged with argon, and the resulting mixture was vigorously stirred under continuous blue light irradiation (Kessil PR160L, λ_{max} = 440 nm) for 16 hours. During the process, a constant flow of argon was maintained using a balloon. After this period, the LED lamp was switched off and MeCN was removed using a rotary evaporator, followed by vacuum drying. The crude product was purified by column chromatography on silica gel, employing hexane/ethyl acetate (98:2) as the eluent.

2. Radical scavenger experiments

In a test tube, 2-allylphenol **1a** (0.3 mmol), diphenyl diselenides **2a** (0.2 mmol), MeCN (2.0 mL) and the radical scavenger reagent (2,2,6,6-tetramethylpiperidine 1-oxyl, TEMPO, 4 equiv, 1.2 mmol) (1,1-diphenyllethylene, DPE, 2 equiv, 0.6 mmol) were mixed up. The vial was then purged with argon, and the resulting mixture was vigorously stirred under continuous blue light irradiation (Kessil PR160L, λ_{max} = 440 nm) for 16 hours. During the process, a constant flow of argon was maintained using a balloon. After this period, the LED lamp was switched off and MeCN was removed using a rotary evaporator, followed by vacuum drying. At the end of the process, the crude mixture was analyzed by GC-MS. No TEMPO-adduct was observed, while the formation of a DPE-adduct was detected (*m/z* = 336) (Figure S1).

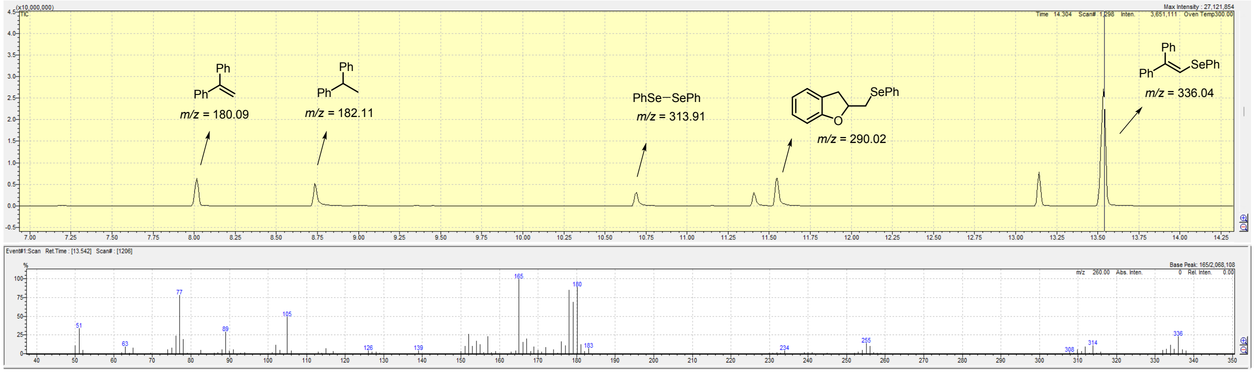

Figure S1. GC-MS analysis of the crude mixture from the reaction performed in the presence of DPE.

3. Visible light photoreaction setup

Reactions were conducted inside a custom wooden box (40 × 40 × 40 cm) equipped with a lower fan supplying cooler external air and an upper fan expelling warmer internal

air. A blue light lamp (Kessil PR160L, λ_{max} = 440 nm) was employed as the irradiation source (Figure S1), with the reaction tube placed 2 cm from the lamp. The internal temperature of the box was maintained at approximately 25 °C throughout the process by continuous air exchange provided by the fans.

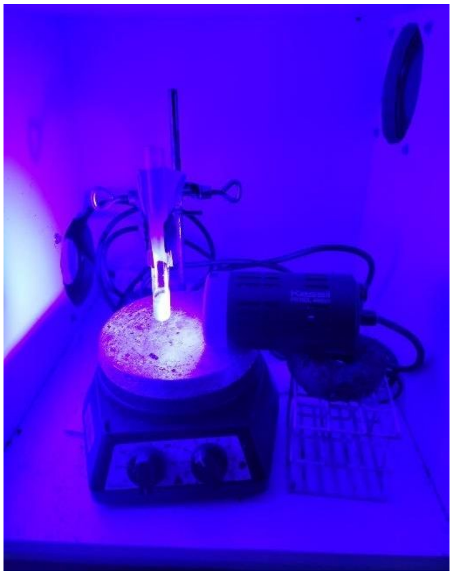

Figure S2. Visible-light setup (Kessil PR160L, λ_{max} = 440 nm) used in this work.

4. UV-Vis analysis of compounds 1a and 2a

The UV-Vis absorption spectra of compounds **1a** and **2a** were recorded with the sample dissolved in MeCN solution against a blank of the same solvent. The spectra were acquired using a Shimadzu UV2600 spectrophotometer at every 1.0 nm in the 300-450 nm window. For this comparative study, the same equivalence was imposed on the derivatives **1a** and **2a** in isolation, as well as in the equimolar mixture of **1a** and **2a**.

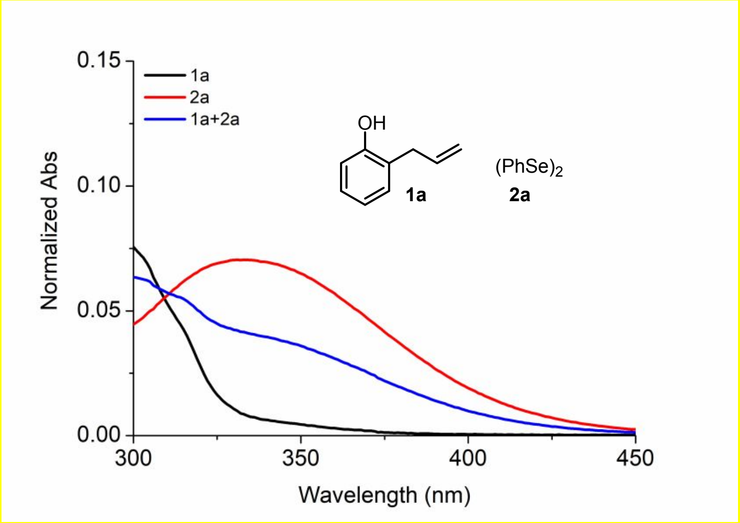

Figure S3. UV-Vis analysis of compounds **1a** and **2a**.

5. Spectral data for the compounds 3a-3r

2-[(Phenylselanyl)methyl]-2,3-dihydrobenzofuran **3a**

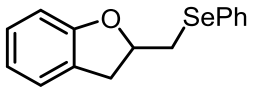

Yield: 0.083 g (96%); yellow solid, mp: 62–63 °C, Lit.[1]: 62–63 °C. **^{1}H NMR** (400 MHz, $CDCl_3$) δ 7.55 – 7.53 (m, 2H), 7.26 – 7.22 (m, 3H), 7.13 – 7.07 (m, 2H), 6.82 (t, *J* = 7.4 Hz, 1H), 6.74 (d, *J* = 8.0 Hz, 1H), 4.96 – 4.89 (m, 1H), 3.37 – 3.29 (m, 2H), 3.11 – 2.98 (m, 2H). **^{13}C NMR** (100 MHz, $CDCl_3$) δ 159.1, 133.0, 129.3, 129.1, 128.0, 127.2, 126.1, 124.9, 120.5, 109.4, 81.8, 35.5, 32.7. **^{77}Se NMR** (76 MHz, $CDCl_3$) δ 256.7.

2-[(*p*-Tolylselanyl)methyl]-2,3-dihydrobenzofuran **3b**

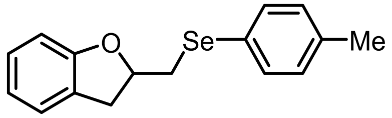

Yield: 0.078 g (86%); yellow solid, mp: 39–41 °C, Lit.[1]: 38–40 °C. **^{1}H NMR** (400 MHz, $CDCl_3$) δ 7.45 (d, *J* = 8.0 Hz, 2H), 7.13 – 7.07 (m, 4H), 6.82 (t, *J* = 7.4 Hz, 1H), 6.74 (d, *J* = 8.1 Hz, 1H), 4.94 – 4.86 (m, 1H), 3.37 – 3.25 (m, 2H), 3.06 – 2.97 (m, 2H), 2.32 (s, 3H). **13C NMR** (100 MHz, $CDCl_3$) δ 159.2, 137.4, 133.6, 130.0, 128.0, 126.2, 125.3, 125.0, 120.5, 109.4, 81.9, 35.4, 33.0, 21.1. **^{77}Se NMR** (76 MHz, $CDCl_3$) δ 249.2.

2-{[(4-Methoxyphenyl)selanyl]methyl}-2,3-dihydrobenzofuran **3c**

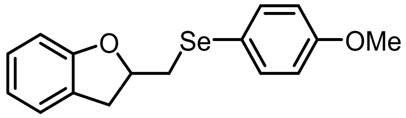

Yield: 0.089 (94%); white solid, mp: 87–88 °C, Lit.[1]: 86–89 °C **^{1}H NMR** (400 MHz, $CDCl_3$) δ 7.51 (d, *J* = 8.9 Hz, 2H), 7.13 – 7.06 (m, 2H), 6.84 – 6.80 (m, 3H), 6.73 (d, *J* = 7.9 Hz, 1H), 4.91 – 4.84 (m, 1H), 3.78 (s, 3H), 3.36 – 3.20 (m, 2H), 3.01 – 2.96 (m, 2H). **^{13}C NMR** (100 MHz, $CDCl_3$) δ 159.5, 159.2, 135.9, 128.0, 126.2, 124.9, 120.4, 119.0, 114.8, 109.4, 81.9, 55.2, 35.4, 33.6. **^{77}Se NMR** (76 MHz, $CDCl_3$) δ 249.1.

2-{[(4-Fluorophenyl)selanyl]methyl}-2,3-dihydrobenzofuran **3d**

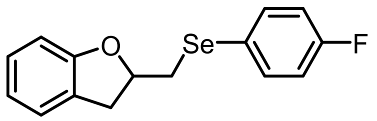

Yield: 0.047 g (51%); yellow solid, 61–62 ºC, Lit.[1]: 59–61 °C. **^{1}H NMR** (400 MHz, $CDCl_3$) δ 7.55 – 7.50 (m, 2H), 7.13 – 7.07 (m, 2H), 6.99 – 6.93 (m, 2H), 6.82 (t, *J* = 7.5 Hz, 1H), 6.72 (d, *J* = 7.9 Hz, 1H), 4.93 – 4.86 (m, 1H), 3.36 – 3.22 (m, 1H), 3.07 – 2.96 (m, 1H). **^{13}C NMR** (100 MHz, CDCl3) δ 162.5 (d, *J* = 247.6 Hz), 159.1, 135.7 (d, *J* = 7.9 Hz), 128.0, 126.0, 124.9, 123.7 (d, *J* = 3.6 Hz), 120.5, 116.3 (d, *J* = 21.5 Hz), 109.4, 81.7, 35.4, 33.5. **^{77}Se NMR** (76 MHz, $CDCl_3$) δ 253.8.

2-{[(4-Chlorophenyl)selanyl]methyl}-2,3-dihydrobenzofuran **3e**

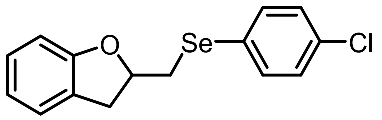

Yield: 0.044 g (45%); yellowish oil. **^{1}H NMR** (400 MHz, $CDCl_3$) δ 7.46 (d, *J* = 8.2 Hz, 1H), 7.22 (d, *J* = 8.2 Hz, 1H), 7.16 – 7.05 (m, 2H), 6.87 – 6.79 (m, 1H), 6.73 (d, *J* = 8.1 Hz, 1H), 4.97 – 4.85 (m, 1H), 3.39 – 3.21 (m, 2H), 3.17 – 2.93 (m, 2H). **^{13}C NMR** (100 MHz, $CDCl_3$) δ 159.0, 134.4, 133.5, 129.3, 128.1, 127.5, 126.0, 124.9, 120.6, 109.4, 81.6, 35.5, 33.1. **^{77}Se NMR** (76 MHz, $CDCl_3$) δ 255.9.

2-{[(3-(Trifluoromethyl)phenyl)selanyl]methyl}-2,3-dihydrobenzofuran **3f**

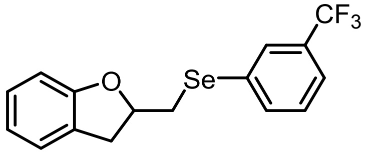

Yield: 0.062 g (58%); yellow oil. **^{1}H NMR** (400 MHz, $CDCl_3$) δ 7.80 (s, 1H), 7.72 (d, *J* = 7.8 Hz, 1H), 7.51 (d, *J* = 7.8 Hz, 1H), 7.38 (t, *J* = 7.9 Hz, 1H), 7.15 – 7.09 (m, 2H), 6.85 (td, *J* = 7.4, 1.1 Hz, 1H), 6.72 (d, *J* = 8.0 Hz, 1H), 5.01 – 4.94 (m, 1H), 3.41 – 3.32 (m, 2H), 3.21 – 3.16 (m, 1H), 3.06 – 3.00 (m, 1H). **^{13}C NMR** (100 MHz, $CDCl_3$) δ 159.0, 135.9, 131.4 (q, *J* = 32.0 Hz), 130.8, 129.4 (q, *J* = 4,3 Hz), 128.2, 125.9, 125.0, 124.0 (q, *J* = 3.8 Hz), 120.7, 109.5, 81.6, 35.6, 33.1. **^{77}Se NMR** (76 MHz, $CDCl_3$) δ 267.1.

5-Methyl-2-[(phenylselanyl)methyl]-2,3-dihydrobenzofuran **3g**

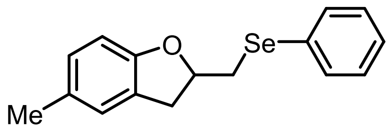

Yield: 0.073 g (80%); White solid, mp: 42–44 ºC, Lit.[2]: 44–45 °C. **^{1}H NMR** (400 MHz, $CDCl_3$) *δ* 7.47 – 7.44 (m, 2H), 7.14 – 7.07 (m, 4H), 6.85 – 6.81 (m, 1H), 6.74 (d, *J* = 7.9 Hz, 1H), 4.94 – 4.87 (m, 1H), 3.38 – 3.26 (m, 2H), 3.07 – 2.98 (m, 2H), 2.33 (s, 3H). **^{13}C NMR** (100 MHz, $CDCl_3$) *δ* 159.2, 137.5, 133.6, 130.0, 128.1, 126.3, 125.4, 125.0, 120.5, 109.5, 82.0, 35.5, 33.0, 21.1. **^{77}Se NMR** (76 MHz, $CDCl_3$) *δ* 249.3.

5-Methyl-2-[(p-tolylselanyl)methyl]-2,3-dihydrobenzofuran **3h**

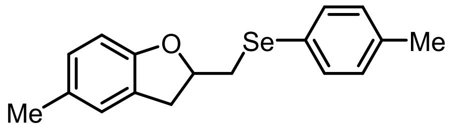

Yield: 0.087 g (92%); yellow oil. **^{1}H NMR** (400 MHz, $CDCl_3$) *δ* 7.38 (d, *J* = 8.2 Hz, 2H), 7.01 (d, *J* = 8.0 Hz, 2H), 6.89 – 6.87 (m, 1H), 6.82 (d, *J* = 7.6 Hz, 1H), 6.58 – 6.55 (m, 1H), 4.84 – 4.77 (m, 1H), 3.26 – 3.16 (m, 2H), 2.98 – 2.87 (m, 2H), 2.25 (s, 3H), 2.19 (s, 3H). **^{13}C NMR** (100 MHz, $CDCl_3$) *δ* 157.1, 137.5, 133.6, 130.0, 129.8, 128.4, 126.3, 125.6, 125.5, 109.0, 82.0, 35.6, 33.1, 21.1, 20.8. **^{77}Se NMR** (76 MHz, $CDCl_3$) *δ* 248.9. HRMS (LCMS Q-TOF) m/z: [M+H]+ calcd for $C_{17}H_{18}OSe$, 319.0595; found, 319.0391.

4-Methyl-2-[(phenylselanyl)methyl]-2,3-dihydrobenzofuran **3i**

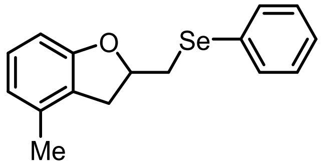

Yield: 0.080 g (88%); yellow solid, mp: 38–40 ºC. **^{1}H NMR** (400 MHz, $CDCl_3$) *δ* 7.56 – 7.54 (m, 2H), 7.27 – 7.25 (m, 3H), 6.97 (d, *J* = 7.5 Hz, 1H), 6.91 (d, *J* = 7.4 Hz, 1H), 6.74 (t, *J* = 7.4 Hz, 1H), 4.98 – 4.90 (m, 1H), 3.39 – 3.32 (m, 2H), 3.12 – 3.0 (m, 2H), 2.14 (s, 3H). **^{13}C NMR** (100 MHz, $CDCl_3$) *δ* 157.6, 133.0, 129.5, 129.2, 129.1, 127.2, 125.3, 122.3, 120.4, 119.6, 81.6, 35.8, 32.8, 15.2. **^{77}Se NMR** (76 MHz, $CDCl_3$) *δ* 258.3. HRMS (LCMS Q-TOF) m/z: [M+H]+ calcd for $C_{16}H_{16}OSe$, 305.0440; found, 305.0441.

5-Chloro-2-[(phenylselanyl)methyl]-2,3-dihydrobenzofuran **3j**

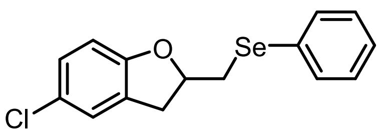

Yield: 0.041 g (42%); yellow solid, mp: 48–50 °C, Lit.[3]: 47–50 °C. **^{1}H NMR** (400 MHz, CDCl3) *δ* 7.55 – 7.53 (m, 2H), 7.28 – 7.26 (m, 3H), 7.09 (d, J = 2.4 Hz, 1H), 7.04 (dd, J = 8.4, 2.4 Hz, 1H), 6.64 (d, J = 8.4 Hz, 1H), 4.99 – 4.92 (m, 1H), 3.36 – 3.28 (m, 2H), 3.11 – 2.98 (m, 2H). **^{13}C NMR** (100 MHz, $CDCl_3$) *δ* 157.9, 133.2, 129.3, 128.2, 127.9, 127.5, 125.2, 125.1, 110.4, 82.6, 35.4, 32.6, 29.7. **^{77}Se NMR** (76 MHz, $CDCl_3$) *δ* 256.2.

5-Chloro-2-[(p-tolylselanyl)methyl]-2,3-dihydrobenzofuran **3k**

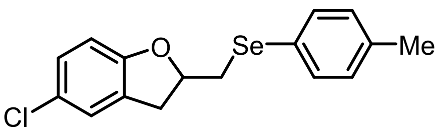

Yield: 0.030 g (30%); yellowish solid, mp: 42-44 ºC. **^{1}H NMR** (400 MHz, $CDCl_3$) *δ* 7.46 – 7.43 (m, 2H), 7.10 – 7.08 (m, 3H), 7.06 – 7.03 (m, 1H), 6.64 (d, *J* = 8.4 Hz, 1H), 4.96 – 4.89 (m, 1H), 3.35 – 3.24 (m, 2H), 3.06 – 2.97 (m, 2H), 2.33 (s, 3H). **^{13}C NMR** (100 MHz, $CDCl_3$) *δ* 157.9, 137.6, 133.7, 130.1, 128.2, 127.9, 125.2, 125.2, 125.1, 110.3, 82.7, 35.4, 32.8, 21.1. **^{77}Se NMR** (76 MHz, $CDCl_3$) *δ* 248.8. HRMS (LCMS Q-TOF) m/z: [M+H]+ calcd for $C_{16}H_{15}ClOSe$, 337.9969; found, 337.9975.

5-Chloro-2-{[(4-chlorophenyl)selanyl]methyl}-2,3-dihydrobenzofuran **3l**

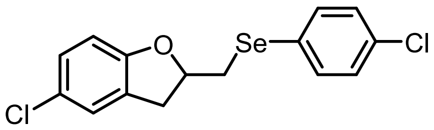

Yield: 0.025 g (23%); yellowish oil. **^{1}H NMR** (400 MHz, $CDCl_3$) *δ* 7.48 – 7.46 (m, 2H), 7.26 – 7.23 (m, 2H), 7.10 – 7.10 (m, 1H), 7.06 – 7.04 (m, 1H), 6.63 (d, *J* = 8.6 Hz, 1H), 4.99 – 4.92 (m, 1H), 3.36 – 3.24 (m, 2H), 3.12 – 2.97 (m, 2H). **^{13}C NMR** (100 MHz, $CDCl_3$) *δ* 157.8, 134.6, 133.8, 133.1, 129.4, 128.0, 127.4, 125.3, 125.1, 110.4, 82.4, 35.4, 33.0. **^{77}Se NMR** (76 MHz, $CDCl_3$) *δ* 255.1. HRMS (LCMS Q-TOF) m/z: [M-H]+ calcd for $C_{15}H_{12}Cl_2OSe$, 356.9353; found, 356.9348.

2-[(Phenylselanyl)methyl]-2,3-dihydronaphtho[2,3-*b*]furan **3m**

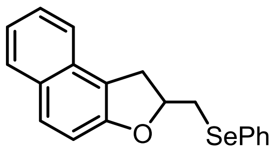

Yield: 0.072 g (66%); white solid, mp: 69-70 ºC, Lit.[2]: 69–70 °C. **^{1}H NMR** (400 MHz, $CDCl_3$) δ 7.72 (d, J = 8.2 Hz, 1H), 7.59 (d, J = 8.7 Hz, 1H), 7.51 – 7.48 (m, 2H), 7.40 – 7.38 (m, 1H), 7.25 – 7.18 (m, 5H), 6.99 (d, J = 8.8 Hz, 1H), 5.10 – 5.03 (m, 1H), 3.59 – 3.53 (m, 1H), 3.35 – 3.20 (m, 2H), 3.12 – 3.07 (m, 1H). **^{13}C NMR** (100 MHz, $CDCl_3$) δ 156.7, 133.2, 129.3, 129.3, 129.3, 129.1, 128.7, 127.4, 126.7, 123.0, 122.7, 117.8, 112.1, 82.7, 34.5, 33.0. **^{77}Se NMR** (76 MHz, $CDCl_3$) δ 256.3.

2-[(*p*-Tolylselanyl)methyl]-2,3-dihydronaphtho[2,3-*b*]furan **3n**

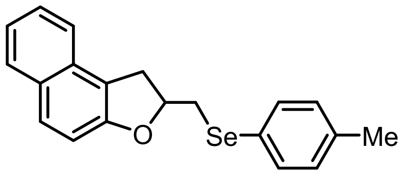

Yield: 0.081 g (72%); yellowish solid, mp: 70-71 ºC. **^{1}H NMR** (400 MHz, $CDCl_3$) δ 7.71 (d, J = 8.2 Hz, 1H), 7.59 (d, J = 8.9 Hz, 1H), 7.47 (d, J = 9.3 Hz, 1H), 7.42 – 7.36 (m, 3H), 7.24 – 7.17 (m, 1H), 7.03 – 6.98 (m, 3H), 5.07 – 4.99 (m, 1H), 3.58 – 3.52 (m, 1H), 3.31 – 3.19 (m, 2H), 3.06 – 3.01 (m, 1H), 2.26 (s, 3H). **^{13}C NMR** (100 MHz, $CDCl_3$) δ 156.7, 137.5, 133.6, 130.8, 130.0, 129.2, 129.0, 128.7, 126.6, 125.3, 122.9, 122.6, 117.8, 112.1, 82.6, 34.4, 33.3, 21.1. **^{77}Se NMR** (76 MHz, $CDCl_3$) δ 248.6. HRMS (LCMS Q-TOF) m/z: [M+H]+ calcd for $C_{20}H_{18}OSe$, 355.0596; found, 355.0595.

2-{[(4-Chlorophenyl)selanyl]methyl}-2,3-dihydronaphtho[2,3-*b*]furan **3o**

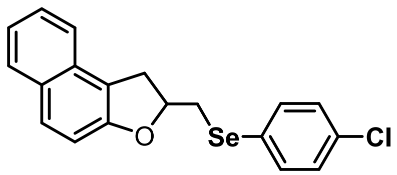

Yield: 0.046 g (39%); yellow oil. **^{1}H NMR** (400 MHz, $CDCl_3$) *δ* 7.79 (d, *J* = 8.2 Hz, 1H), 7.66 (d, *J* = 8.8 Hz, 1H), 7.54 – 7.45 (m, 4H), 7.32 – 7.29 (m, 1H), 7.23 – 7.21 (m, 2H), 7.04 (d, *J* = 8.8 Hz, 1H), 5.15 – 5.08 (m, 1H), 3.64 – 3.58 (m, 1H), 3.37 – 3.32 (m, 1H), 3.29 – 3.24 (m, 1H), 3.18 – 3.13 (m, 1H). **^{13}C NMR** (100 MHz, $CDCl_3$) *δ* 156.6, 134.5, 133.6, 130.7, 129.3, 129.3, 129.1, 128.7, 127.5, 126.7, 122.9, 122.6, 117.6, 112.0, 82.4, 34.4, 33.4. **^{77}Se NMR** (76 MHz, $CDCl_3$) *δ* 255.4.

2-[(Naphthalen-1-ylselanyl)methyl]-2,3-dihydrobenzofuran **3p**

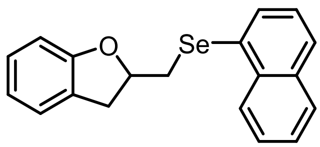

Yield: 0.071 g (65%); yellow solid, mp: 82-85 ºC. **^{1}H NMR** (400 MHz, $CDCl_3$) *δ* 8.35 (d, *J* = 8.4 Hz, 1H), 7.80 – 7.72 (m, 3H), 7.50 – 7.41 (m, 2H), 7.31 – 7.27 (m, 1H), 7.04 – 6.99 (m, 2H), 6.74 (t, *J* = 7.0 Hz, 1H), 6.64 (d, *J* = 8.0 Hz, 1H), 4.83 – 4.76 (m, 1H), 3.29 – 3.21 (m, 2H), 3.04 – 2.91 (m, 2H). **^{13}C NMR** (100 MHz, $CDCl_3$) *δ* 159.1, 134.4, 134.0, 133.3, 128.9, 128.7, 128.4, 128.0, 127.7, 126.8, 126.3, 126.1, 125.7, 125.0, 120.5, 109.5, 81.9, 35.5, 32.9. **^{77}Se NMR** (76 MHz, CDCl3) *δ* 194.0. **HRMS** (LCMS Q-TOF) m/z: [M+H]+ calcd for $C_{19}H_{16}OSe$, 341.0440; found, 341.0446.

2-[(Naphthalen-1-ylselanyl)methyl]-1,2-dihydronaphtho[2,1-*b*]furan **3q**

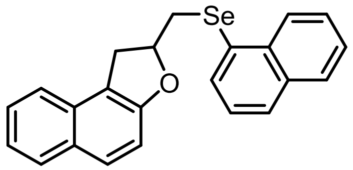

Yield: 0.066 g (59%); yellowish solid, mp: 78-79 °C, Lit.[2]: 78–79 °C. **^{1}H NMR** (400 MHz, $CDCl_3$) *δ* 8.37 (d, J = 8.2 Hz, 1H), 7.86 – 7.81 (m, 1H), 7.81 – 7.70 (m, 3H), 7.63 – 7.54 (m, 2H), 7.51 – 7.44 (m, 3H), 7.42 – 7.35 (m, 1H), 7.30 (t, J = 7.7 Hz, 1H), 7.22 (t, J = 6.8 Hz, 1H), 7.01 – 6.95 (m, 1H), 5.04 – 4.97 (m, 1H), 3.56 – 3.50 (m, 1H), 3.37 – 3.32 (m, 1H), 3.26 – 3.20 (m, 1H), 3.13 – 3.08 (m, 1H). **^{13}C NMR** (100 MHz, $CDCl_3$) *δ* 156.7, 134.0, 133.4, 129.2, 129.0, 128.9, 128.7, 128.7, 127.7, 126.9, 126.7, 126.3, 125.8, 124.6, 123.4, 122.9, 122.6, 117.7, 112.0, 82.6, 34.4, 33.2. **^{77}Se NMR** (76 MHz, $CDCl_3$) *δ* 193.1.

2-[(*p*-Tolylthio)methyl]-2,3-dihydrobenzofuran **3r**

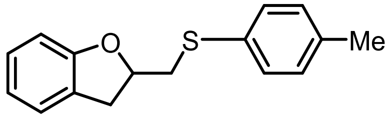

Yield: 0.015 g (20%); yellow oil. **^{1}H NMR** (400 MHz, $CDCl_3$) *δ* 7.34 – 7.31 (m, 2H), 7.16 – 7.08 (m, 4H), 6.88 – 6.75 (m, 2H), 4.90 – 4.83 (m, 1H), 3.46 – 3.30 (m, 2H), 3.08 – 3.02 (m, 2H), 2.32 (s, 3H). **^{13}C NMR** (100 MHz, $CDCl_3$) *δ* 159.2, 136.9, 131.5, 130.8, 129.9, 128.1, 126.1, 125.0, 120.6, 109.5, 81.2, 39.6, 34.9, 21.1.

6. References

1. Bartz, R. H.; Souza, P. S.; Iarocz, L. E. B.; Hellwig, P. S.; Jacob, R. G.; Silva, M. S.; Lenardão, E. J.; Perin, G. *Eur. J. Org. Chem.* **2025,** *28,* e202401243.
2. Scheide, M. R.; Schneider, A. R.; Jardim, G. A. M.; Martins, G. M.; Durigon, D. C.; Saba, S.; Rafique, J.; Braga, A. L. *Org. Biomol. Chem.* **2020,** *18,* 4916–4921.
3. Wang, Y.; Liu, J.; Zhu, Q. 2-Phenylselenomethyl-2,3-dihydrobenzofuran as Monoamine Oxidase Inhibitor and its Preparation. Chin. Patent CN102250048 A, Nov 23, 2011.

7. Selected Spectra

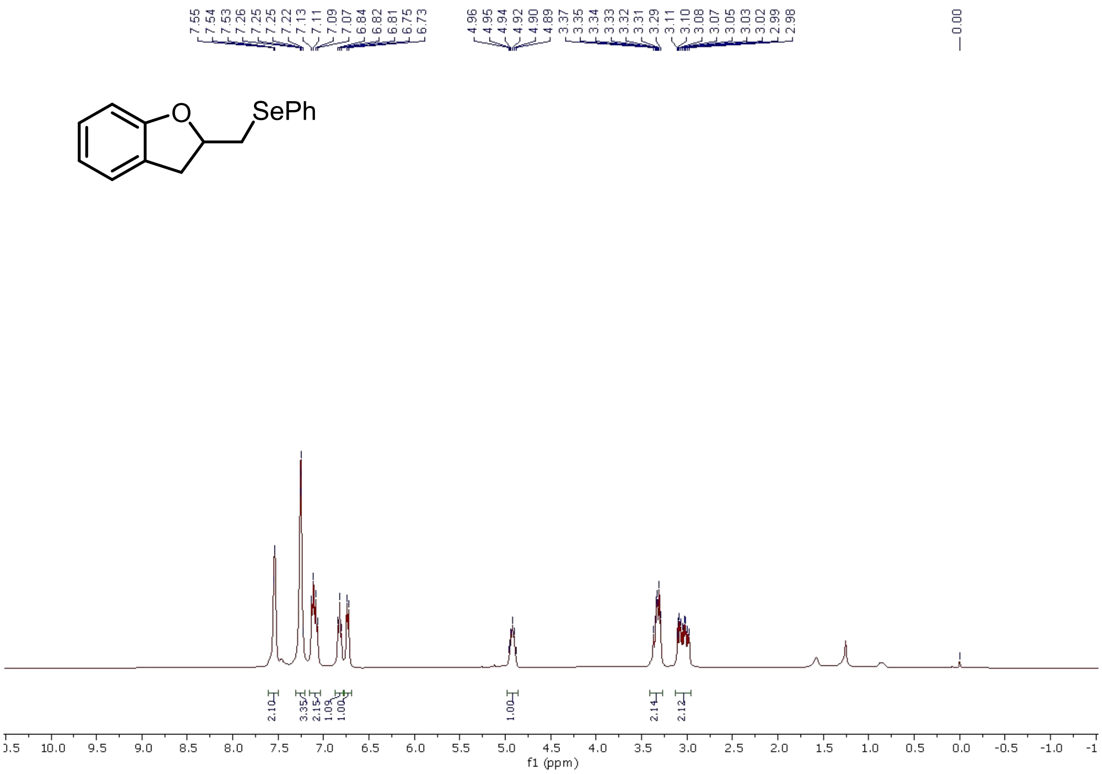

Figure S4. ^{1}H NMR (400 MHz, $CDCl_3$) spectrum of the compound **3a**.

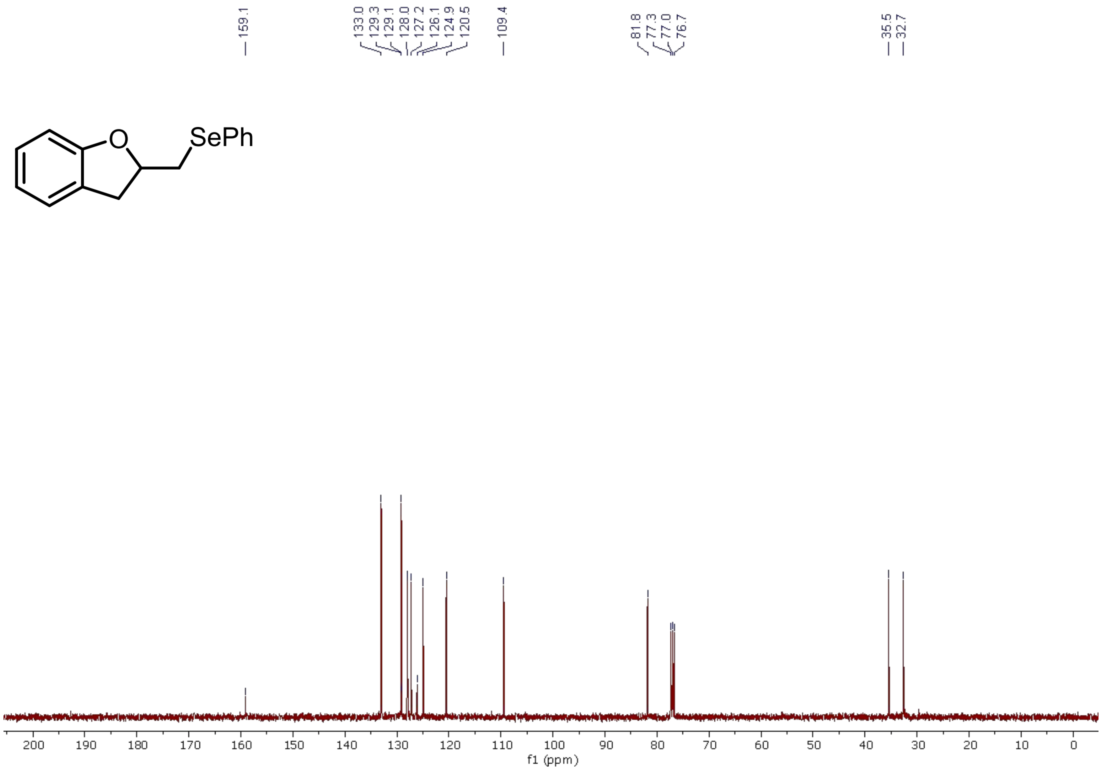

Figure S5. ^{13}C NMR (100 MHz, $CDCl_3$) spectrum of the compound **3a**.

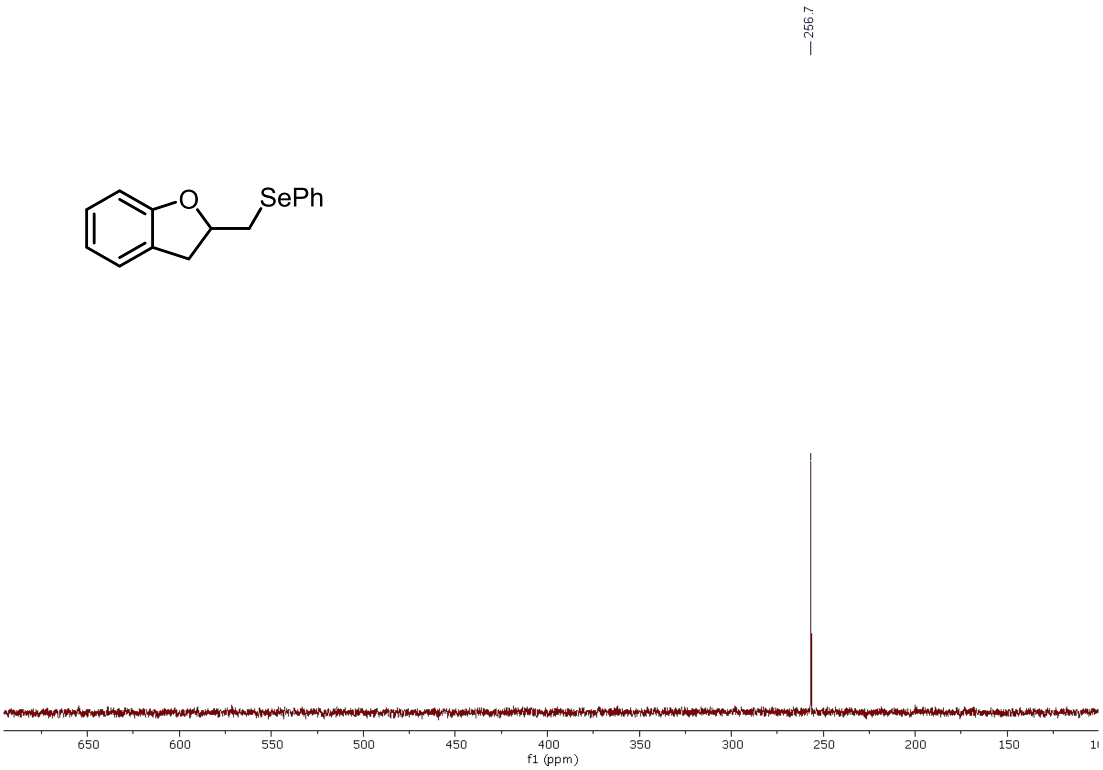

Figure S6. ^{77}Se NMR (100 MHz, $CDCl_3$) spectrum of the compound **3a**.

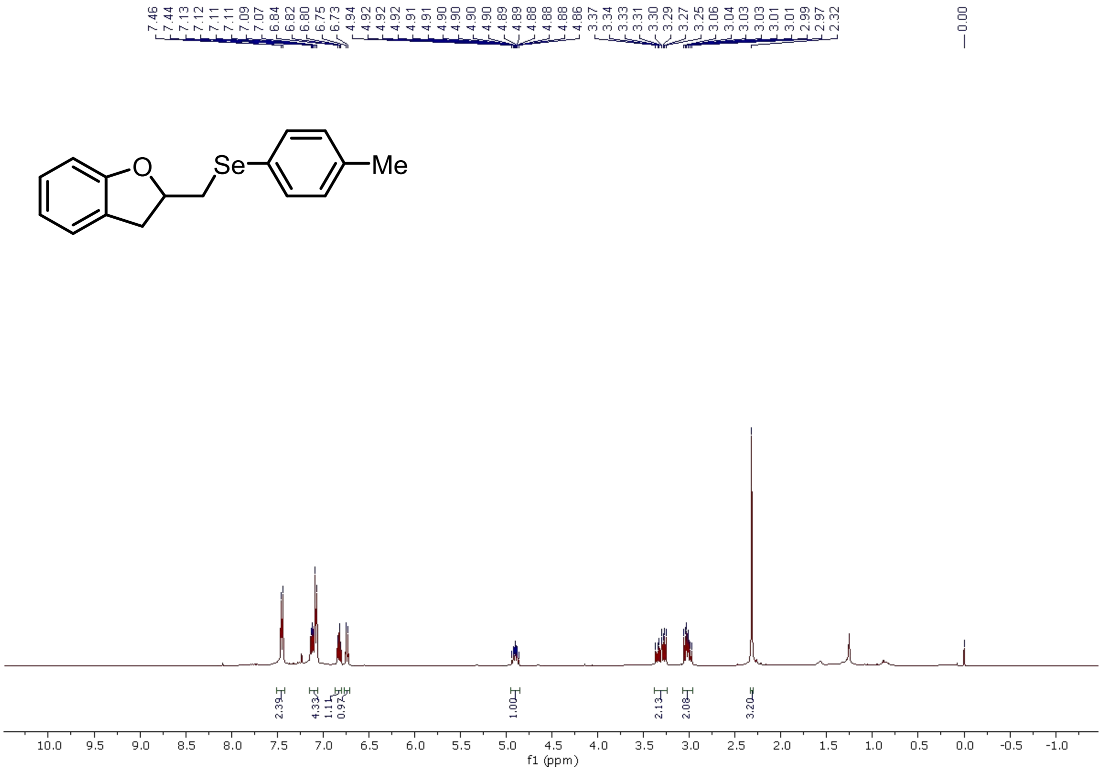

Figure S7. ^{1}H NMR (400 MHz, $CDCl_3$) spectrum of the compound **3b**.

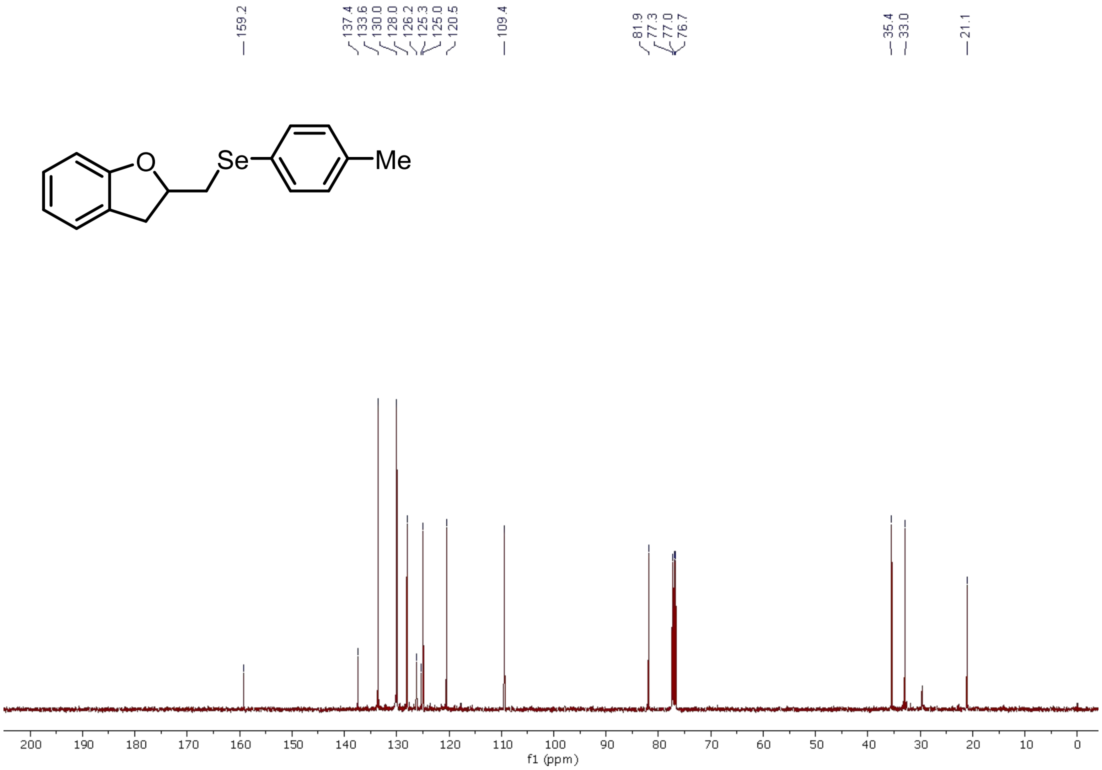

Figure S8. ^{13}C NMR (100 MHz, $CDCl_3$) spectrum of the compound **3b**.

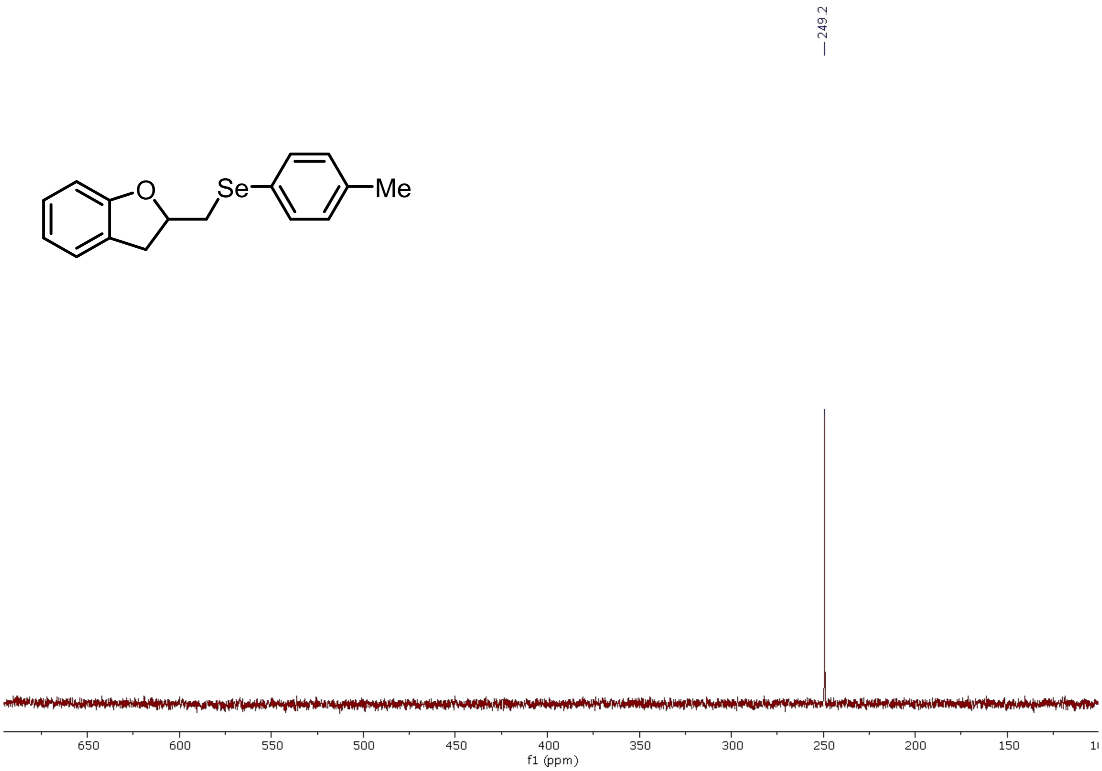

Figure S9. ^{77}Se NMR (100 MHz, $CDCl_3$) spectrum of compound **3b**.

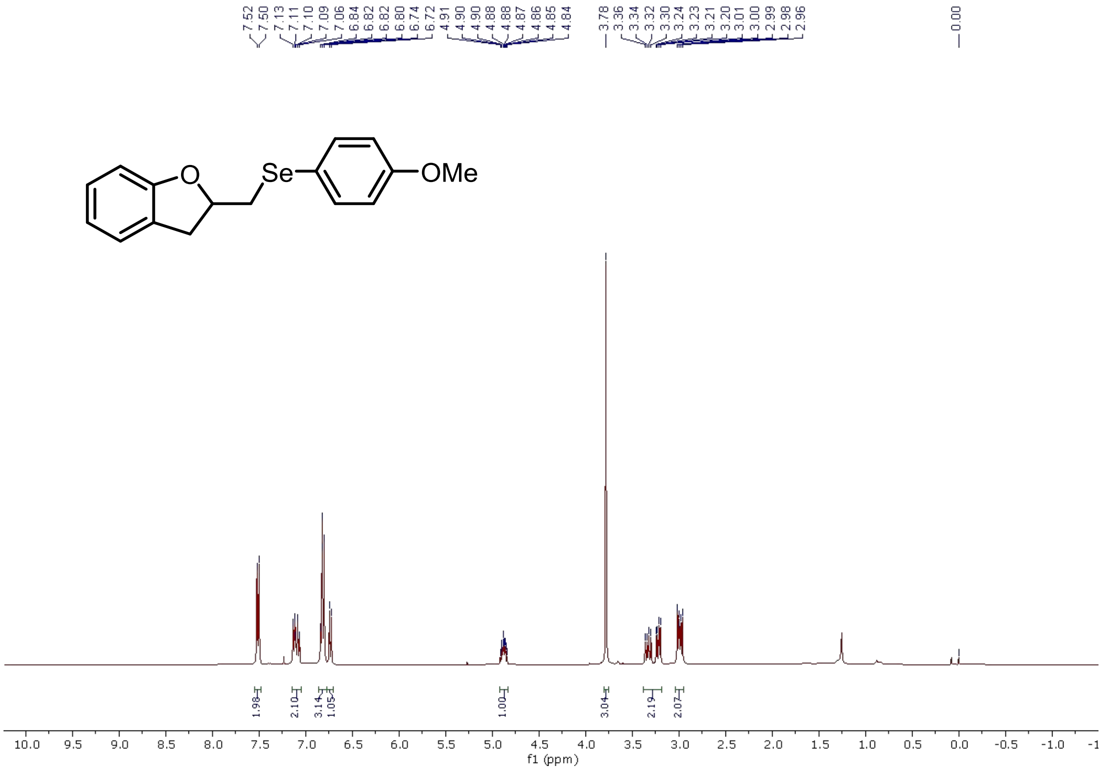

Figure S10. ^{1}H NMR (400 MHz, $CDCl_3$) spectrum of the compound **3c**.

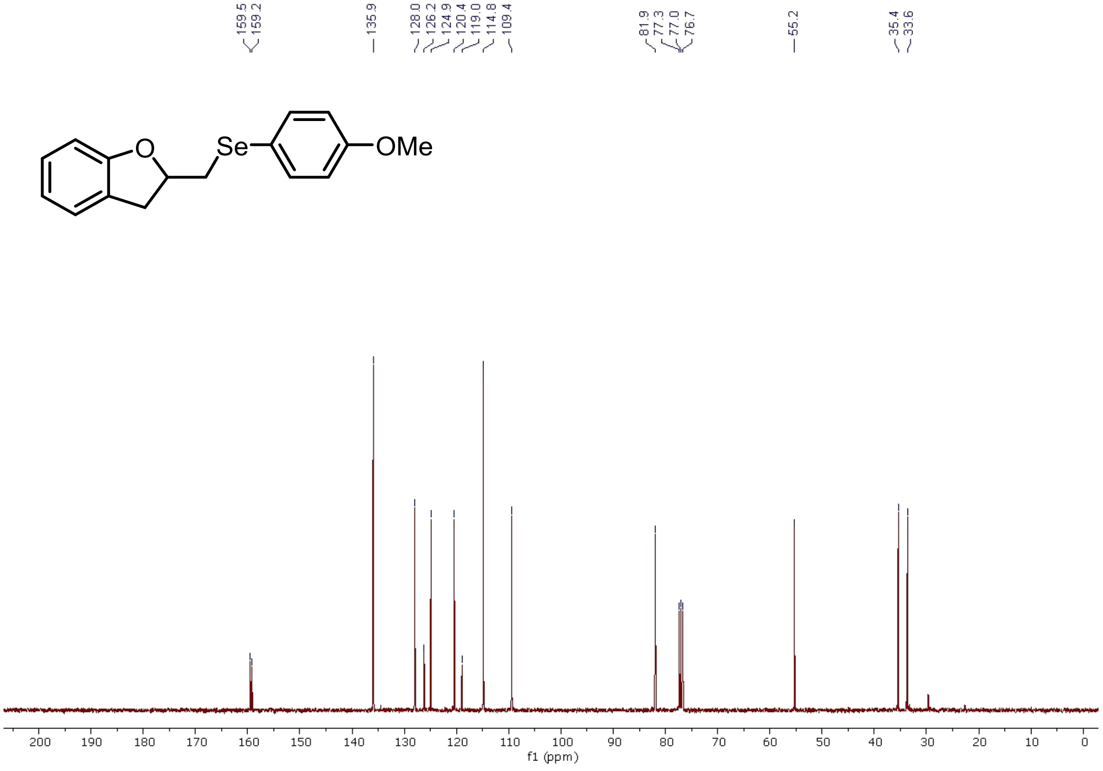

Figure S11. ^{13}C NMR (100 MHz, $CDCl_3$) spectrum of the compound **3c**.

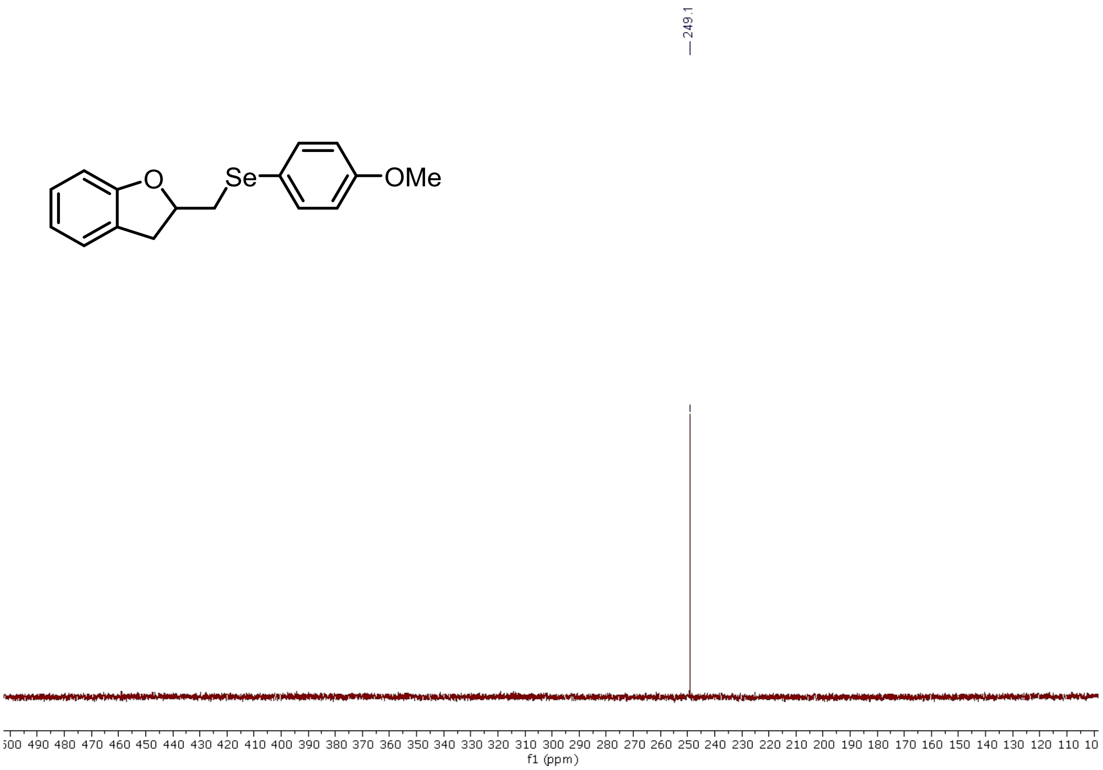

Figure S12. ^{77}Se NMR (100 MHz, $CDCl_3$) spectrum of the compound **3c**.

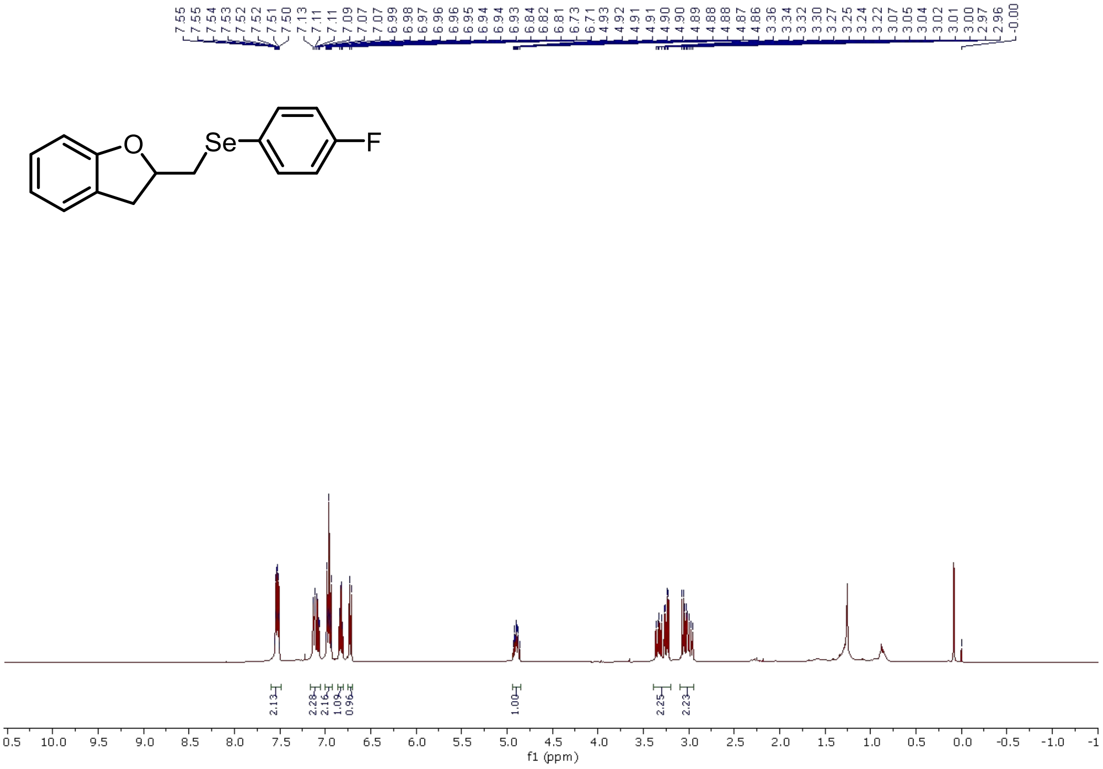

Figure S13. ^{1}H NMR (400 MHz, $CDCl_3$) spectrum of the compound **3d**.

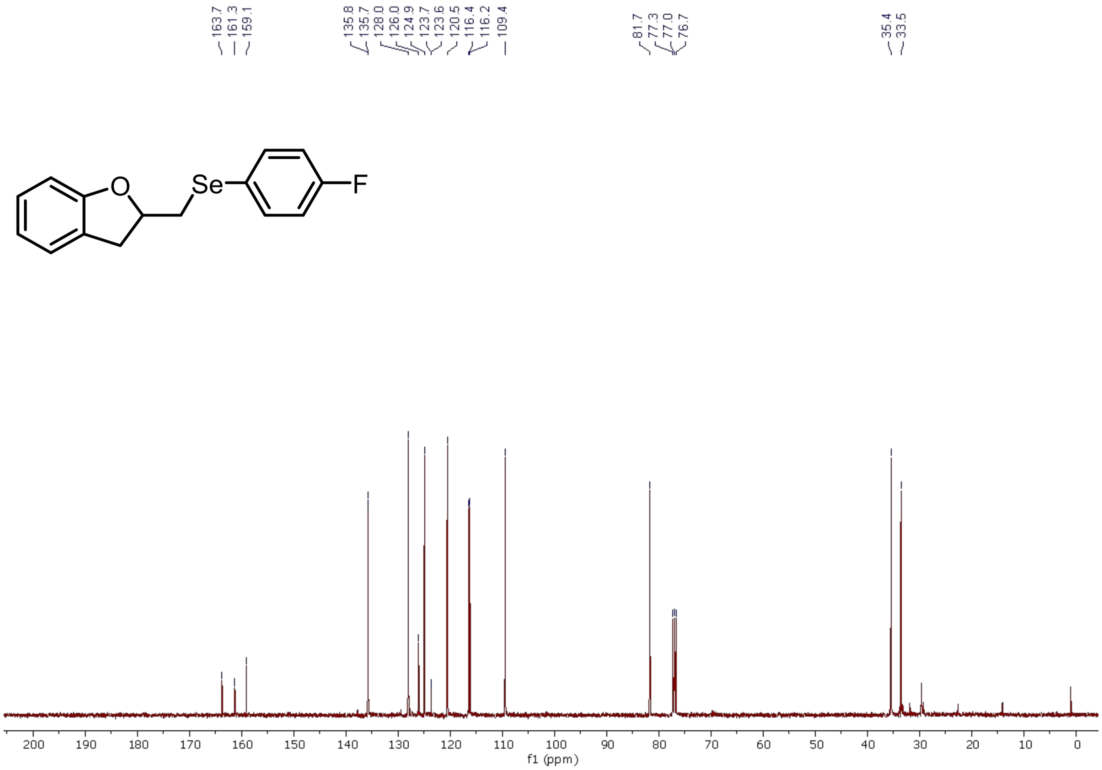

Figure S14. ^{13}C NMR (100 MHz, $CDCl_3$) spectrum of the compound **3d**.

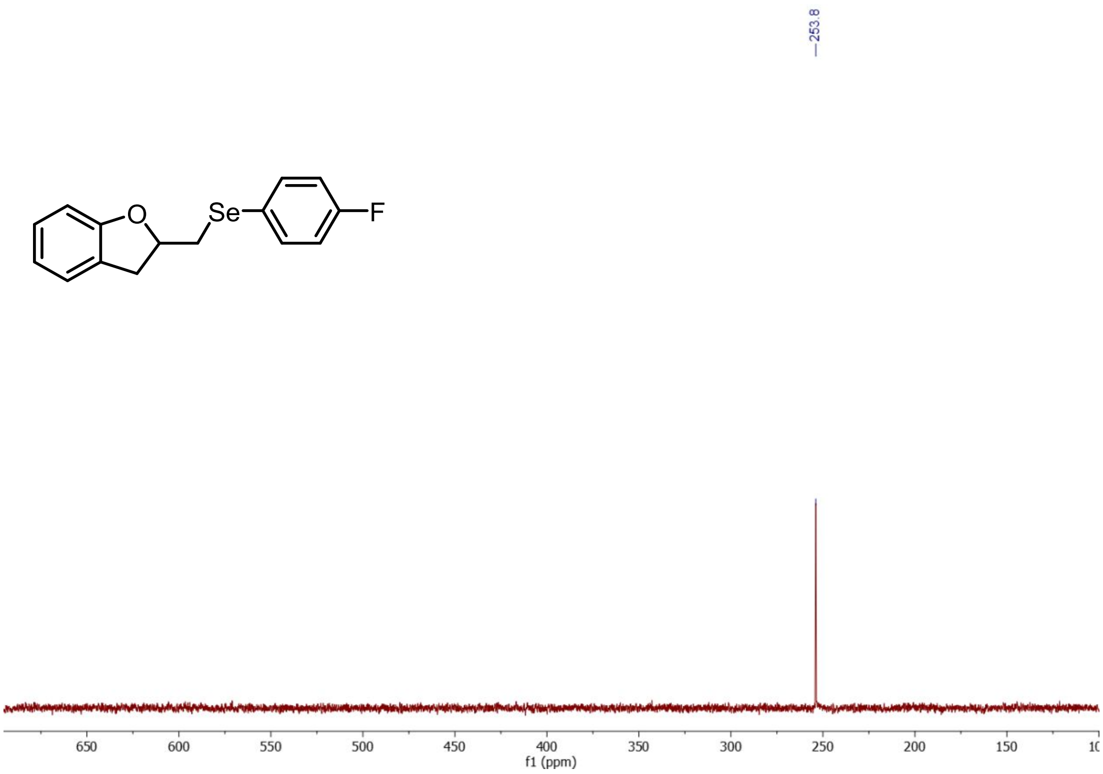

Figure S15. ^{77}Se NMR (100 MHz, $CDCl_3$) spectrum of the compound **3d**.

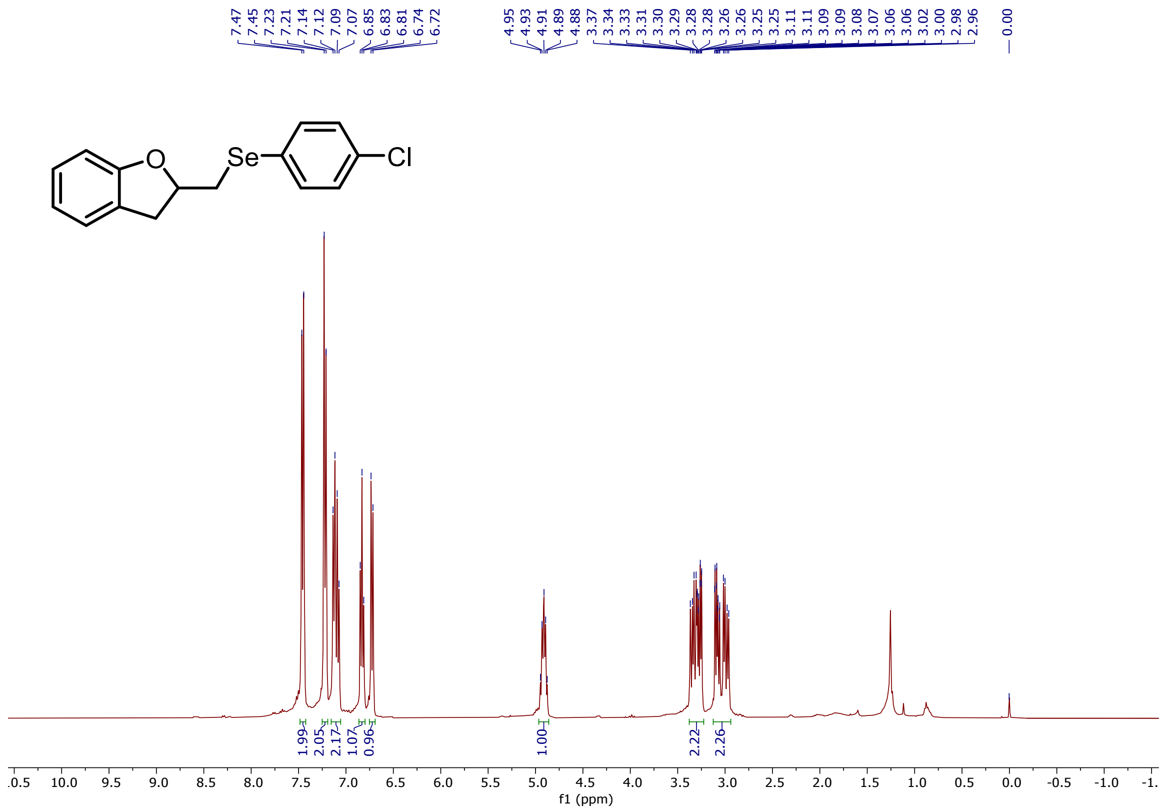

Figure S16. ^{1}H NMR (400 MHz, $CDCl_3$) spectrum of the compound **3e**.

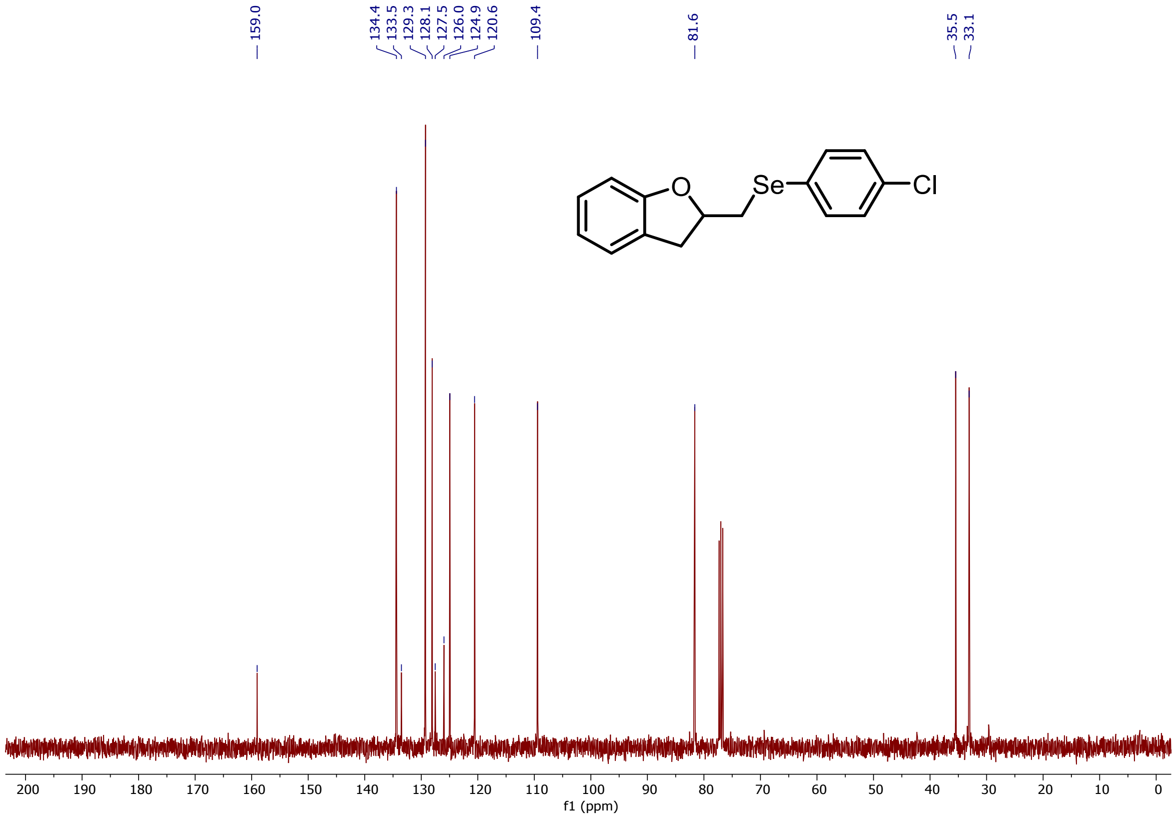

Figure S17. ^{13}C NMR (100 MHz, $CDCl_3$) spectrum of the compound **3e**.

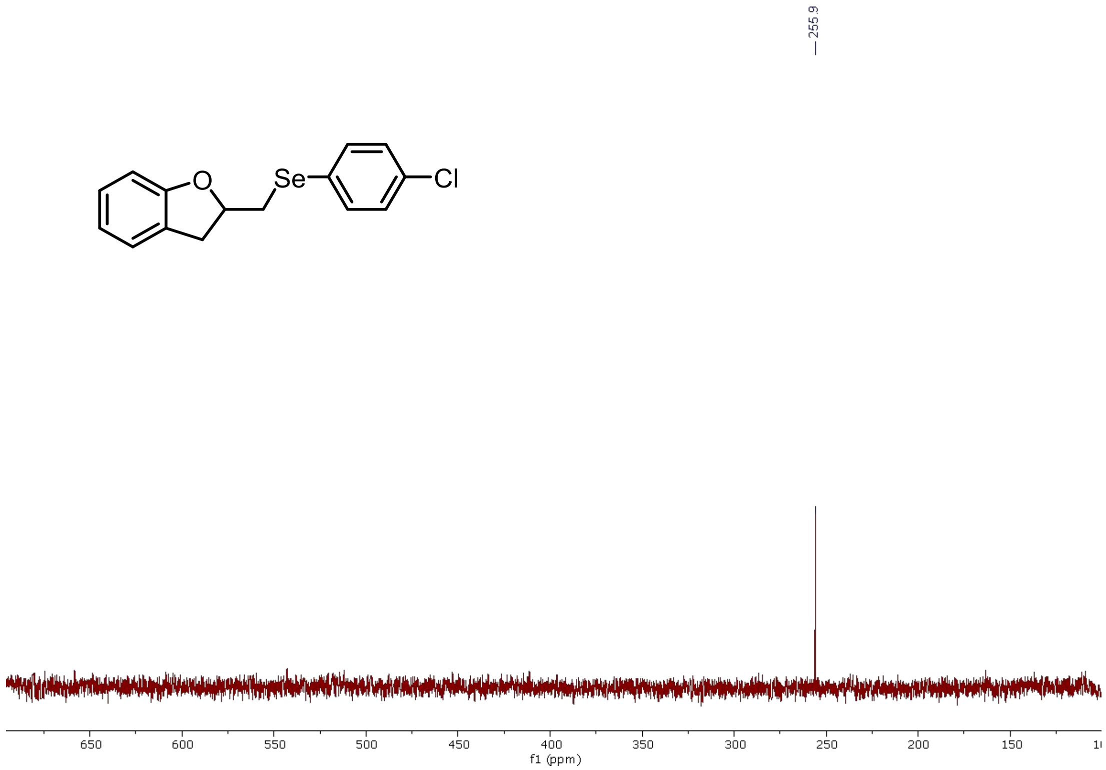

Figure S18. ^{77}Se NMR (100 MHz, $CDCl_3$) spectrum of compound **3e**.

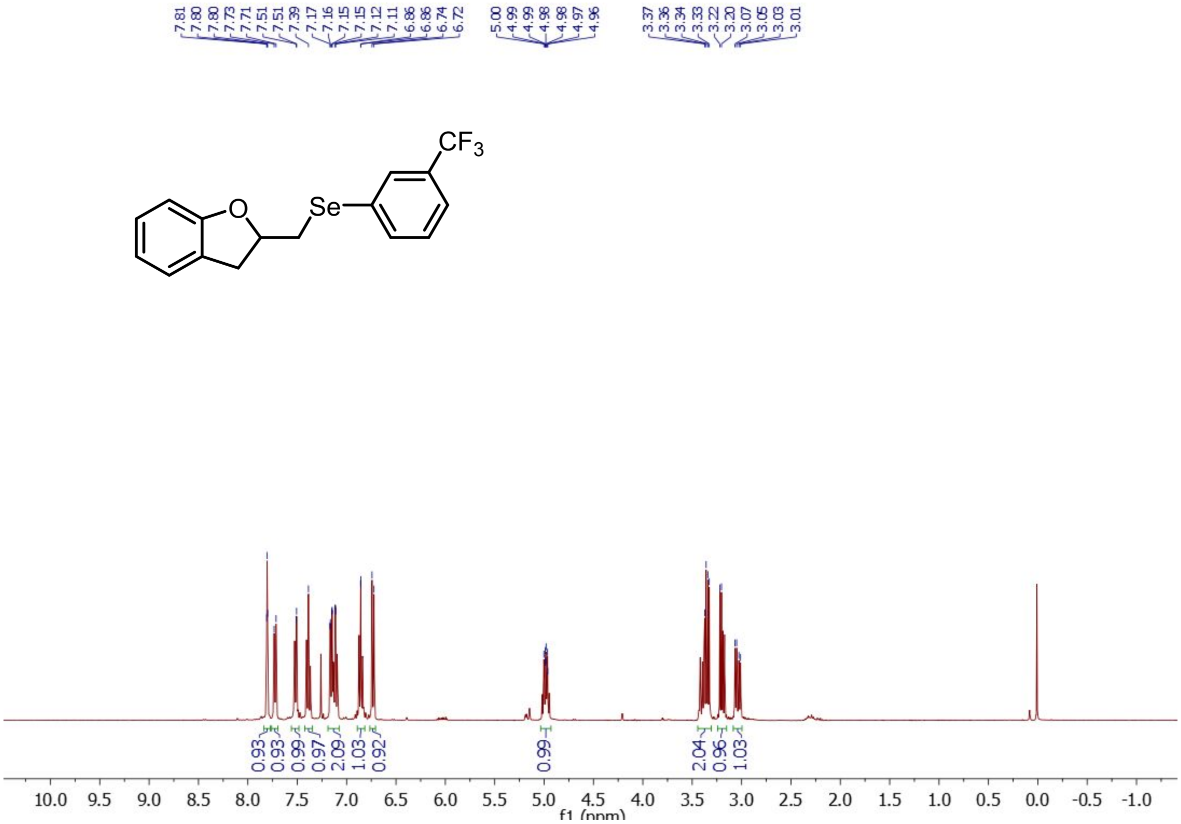

Figure S19. ^{1}H NMR (400 MHz, $CDCl_3$) spectrum of compound **3f**.

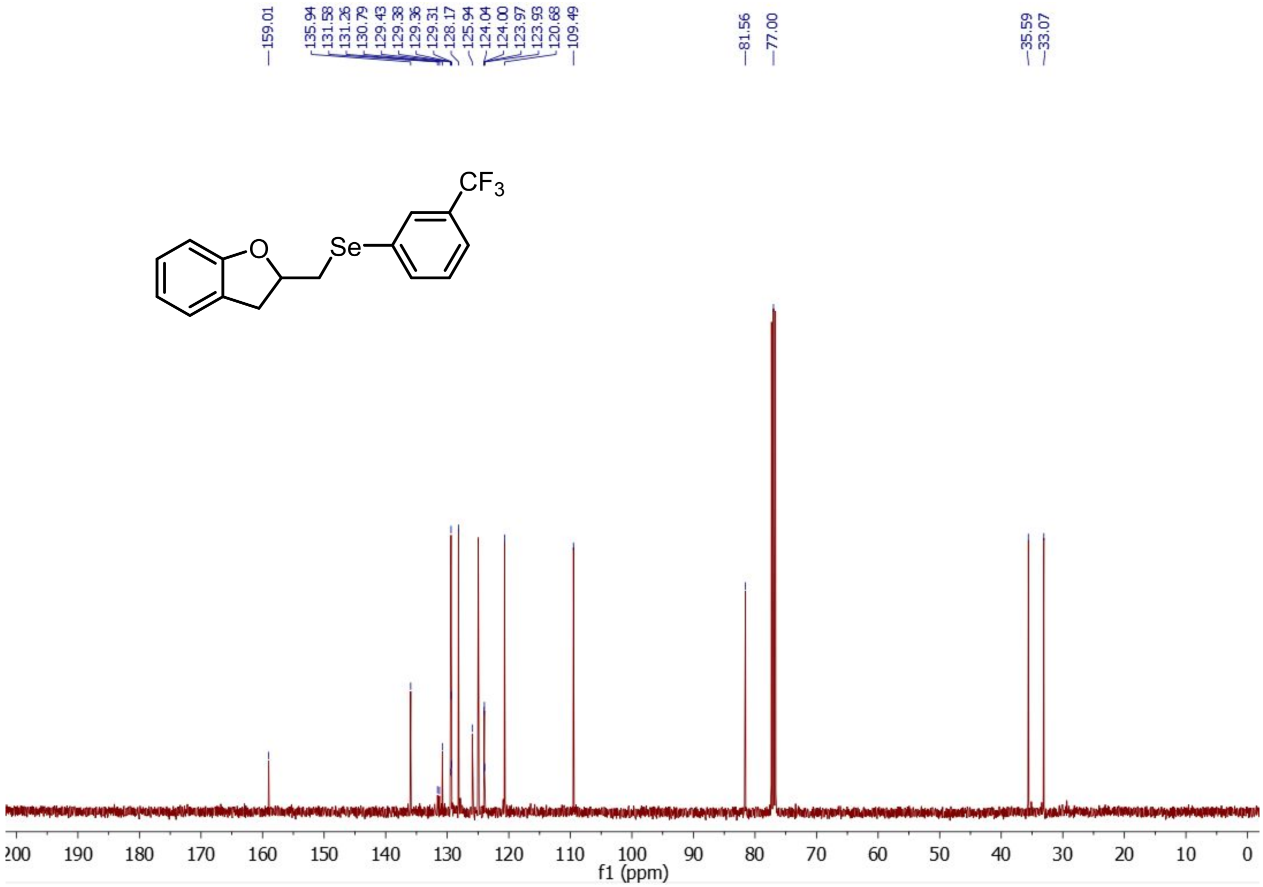

Figure S20. ^{13}C NMR (100 MHz, $CDCl_3$) spectrum of the compound **3f**.

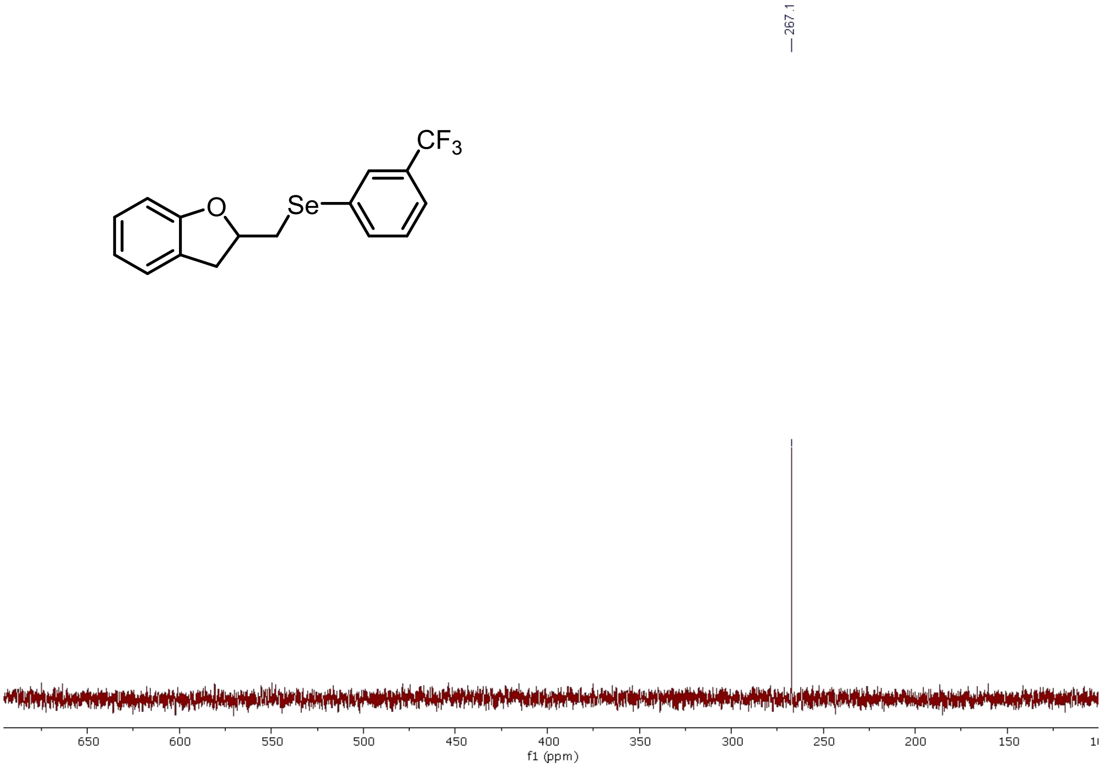

Figure S21. ^{77}Se NMR (100 MHz, $CDCl_3$) spectrum of compound **3f**.

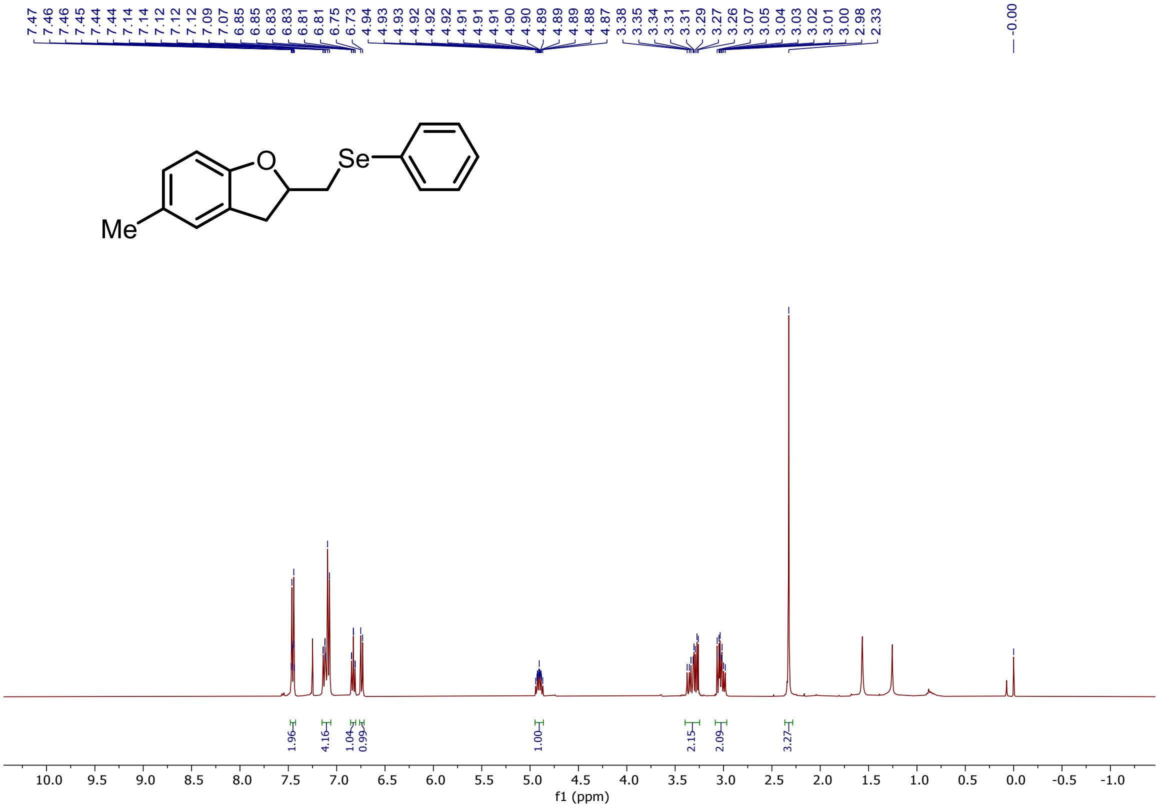

Figure S22. ^{1}H NMR (400 MHz, $CDCl_3$) spectrum of compound **3g**.

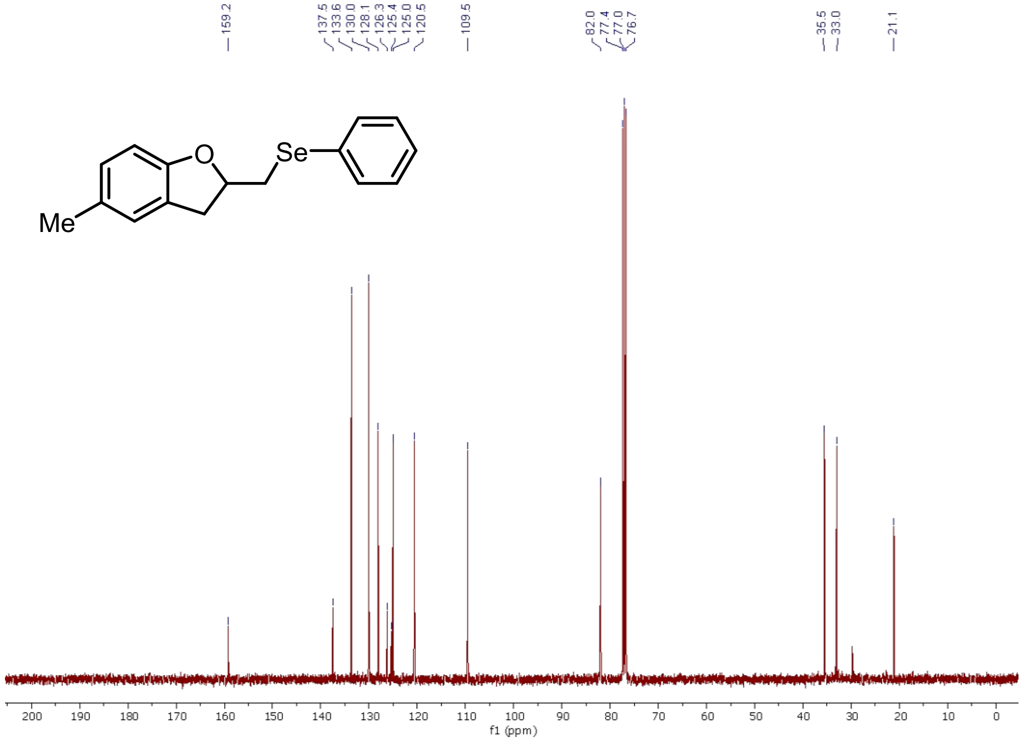

Figure S23. ^{13}C NMR (100 MHz, $CDCl_3$) spectrum of the compound **3g**.

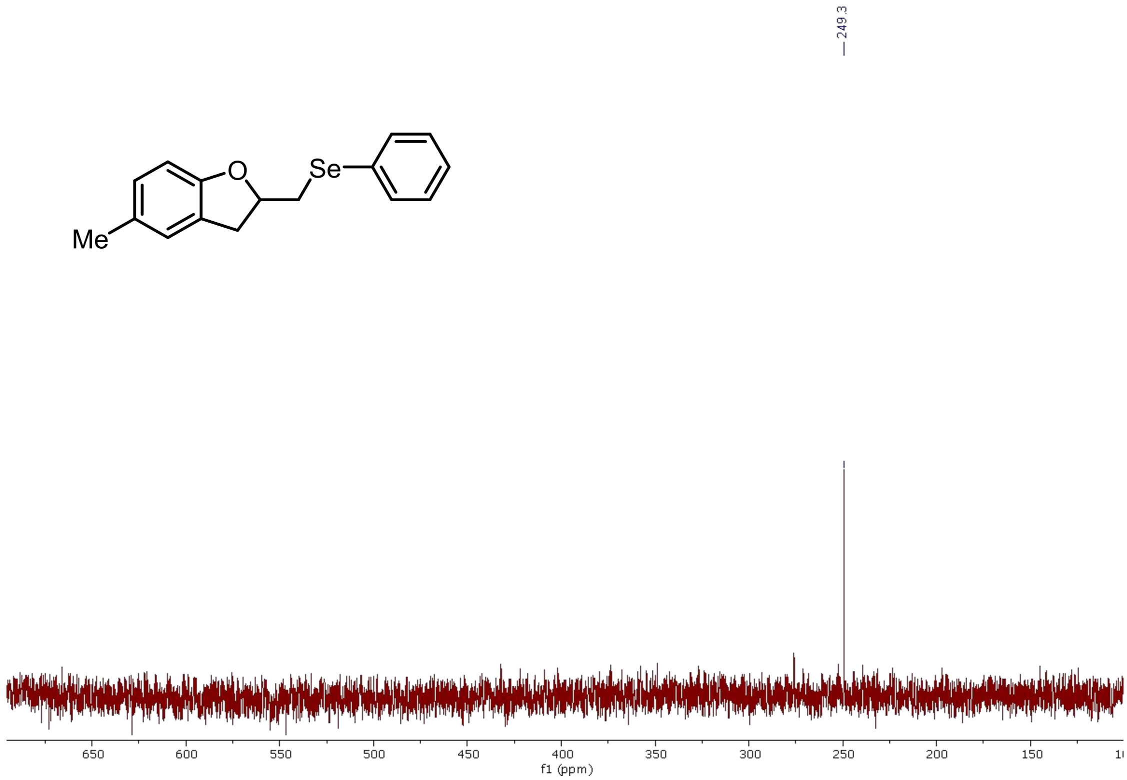

Figure S24. ^{77}Se NMR (100 MHz, $CDCl_3$) spectrum of compound **3g**.

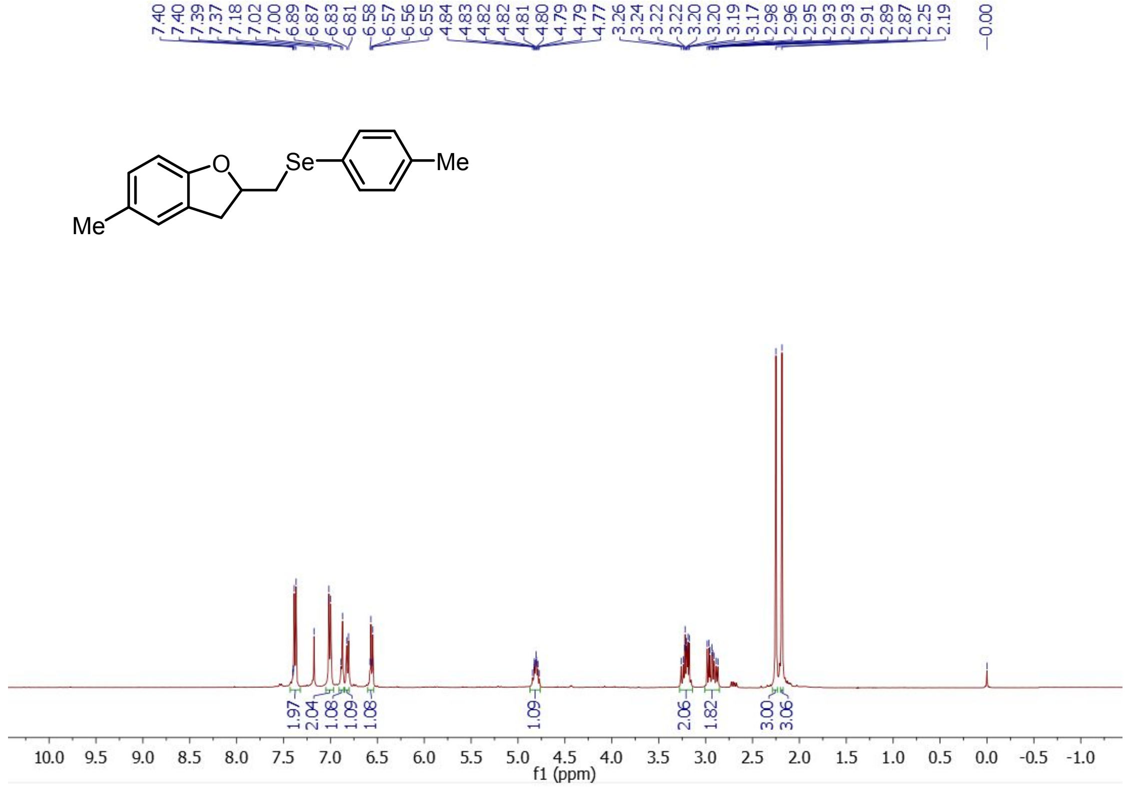

Figure S25. ^{1}H NMR (400 MHz, $CDCl_3$) spectrum of compound **3h**.

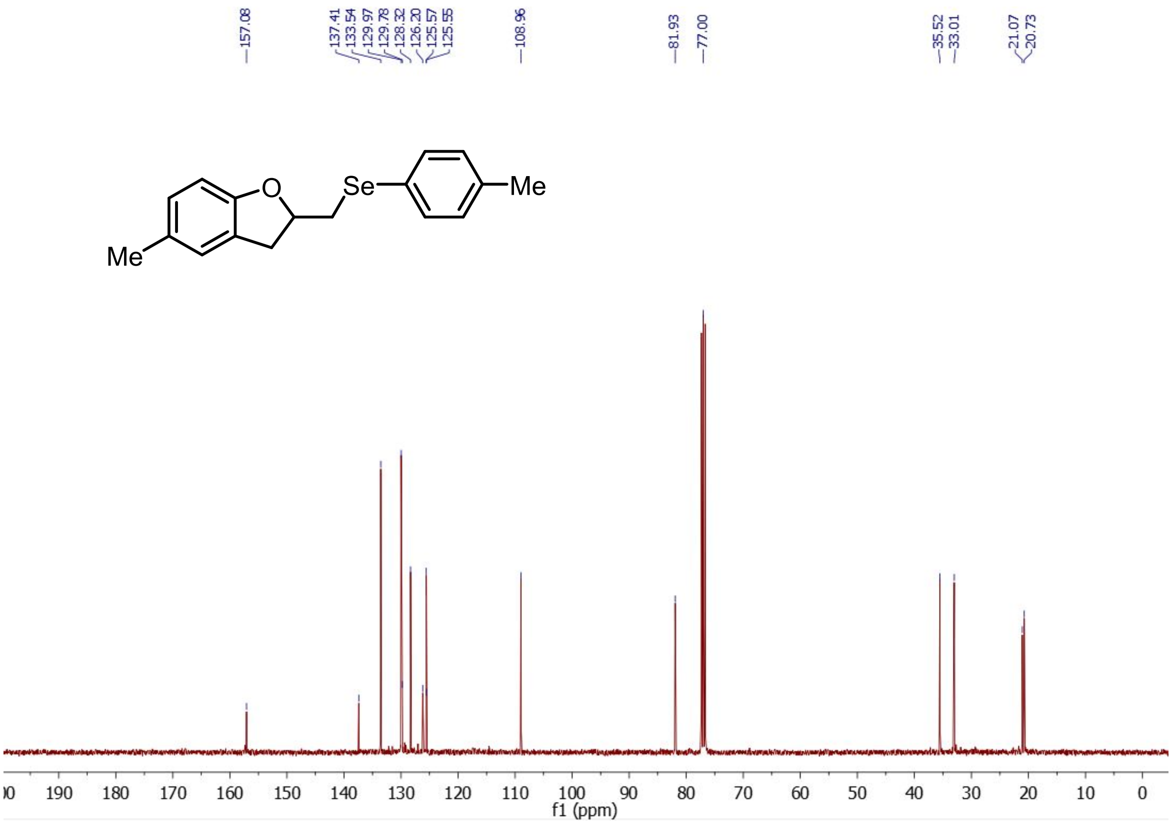

Figure S26. ^{13}C NMR (100 MHz, $CDCl_3$) spectrum of the compound **3h**.

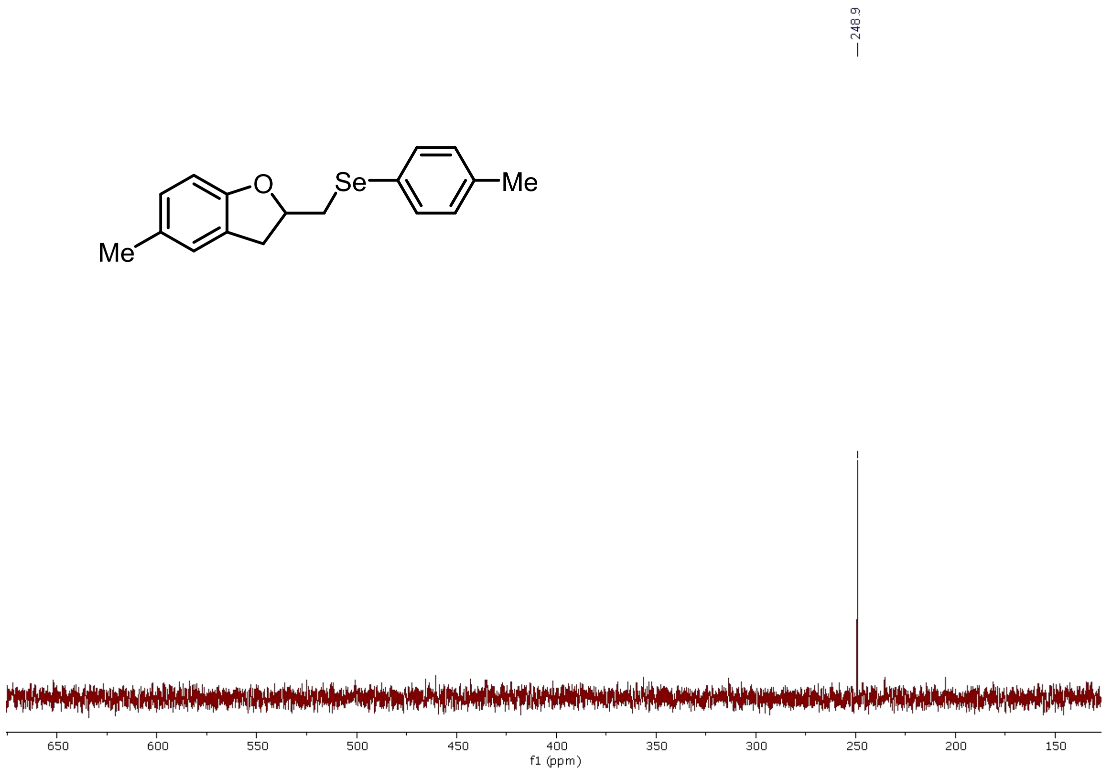

Figure S27. ^{77}Se NMR (100 MHz, $CDCl_3$) spectrum of compound **3h**.

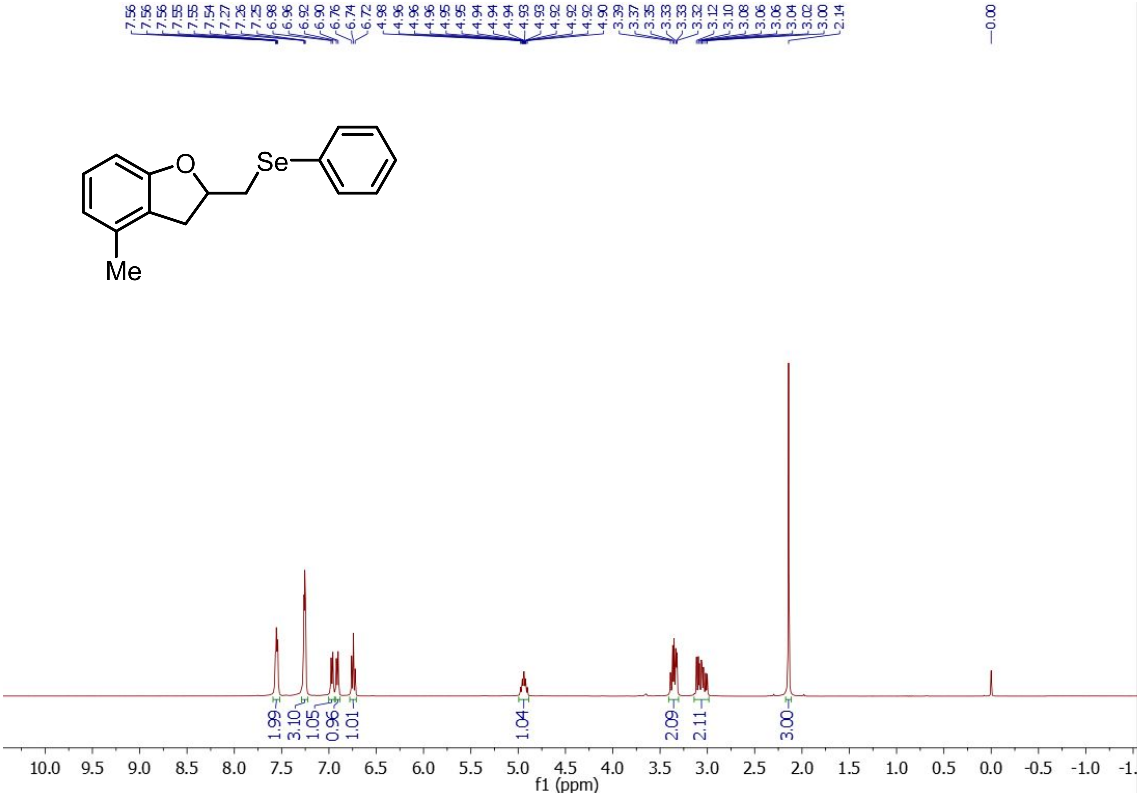

Figure S28. ^{1}H NMR (400 MHz, $CDCl_3$) spectrum of compound **3i**.

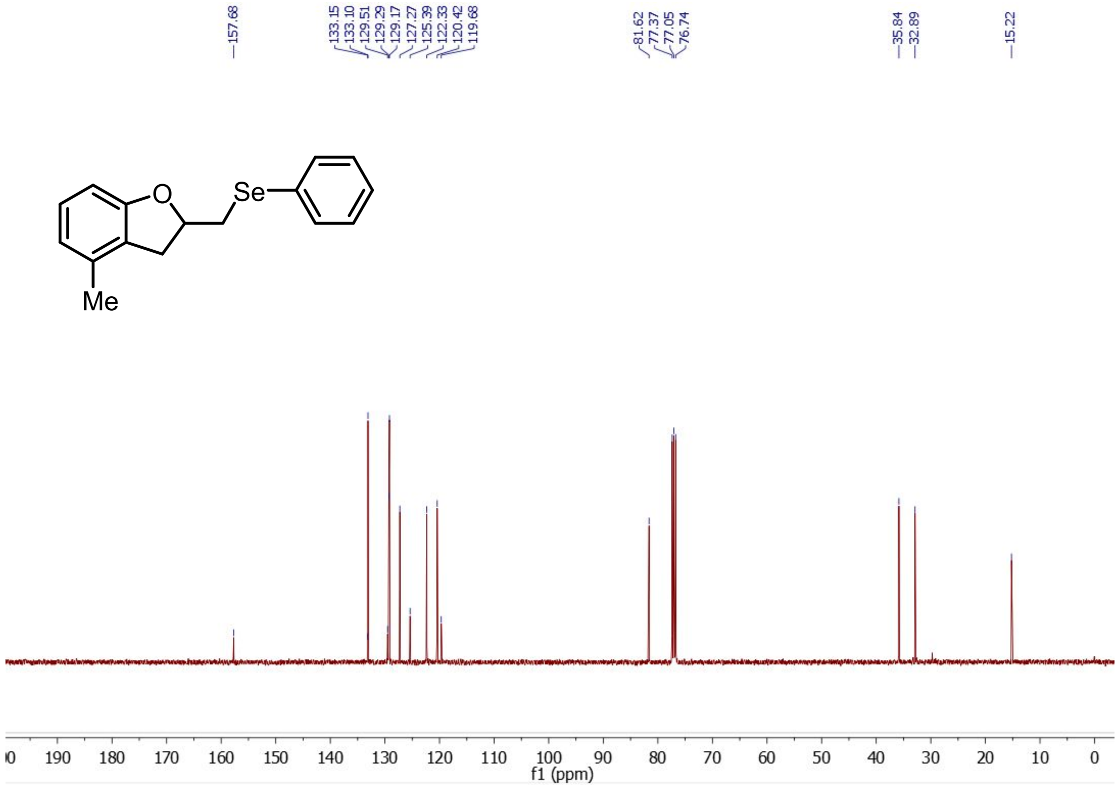

Figure S29. ^{13}C NMR (100 MHz, $CDCl_3$) spectrum of the compound **3i**.

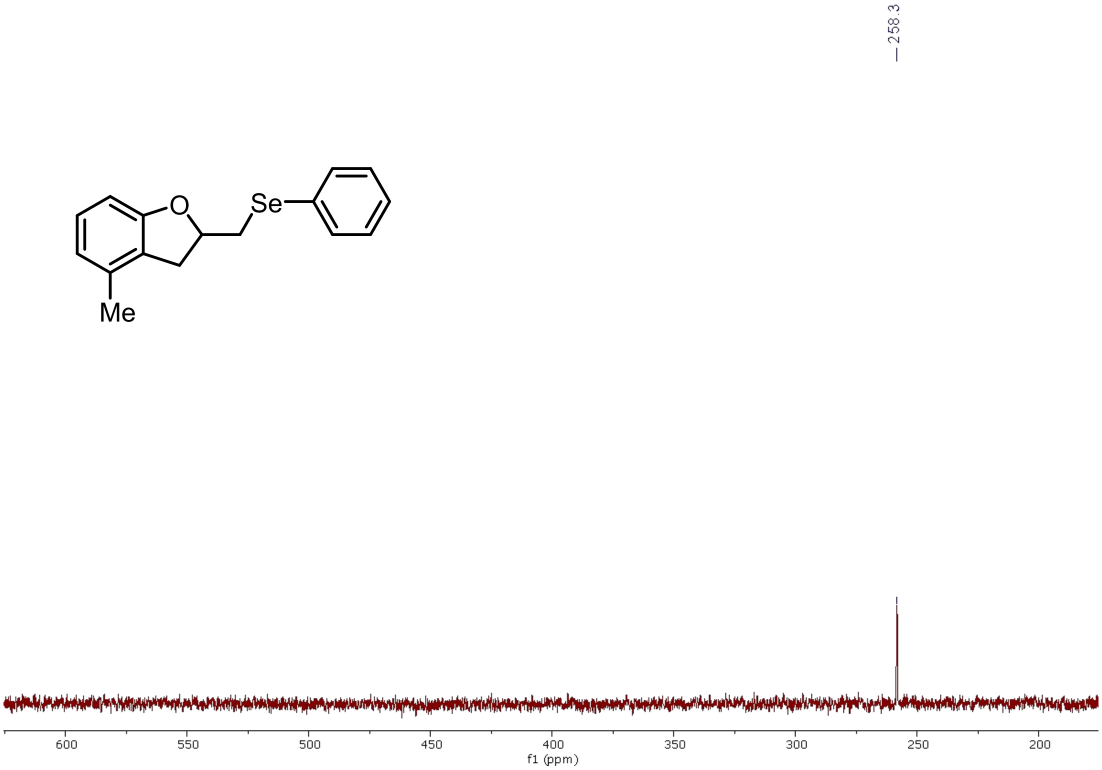

Figure S30. ^{77}Se NMR (100 MHz, $CDCl_3$) spectrum of compound **3i**.

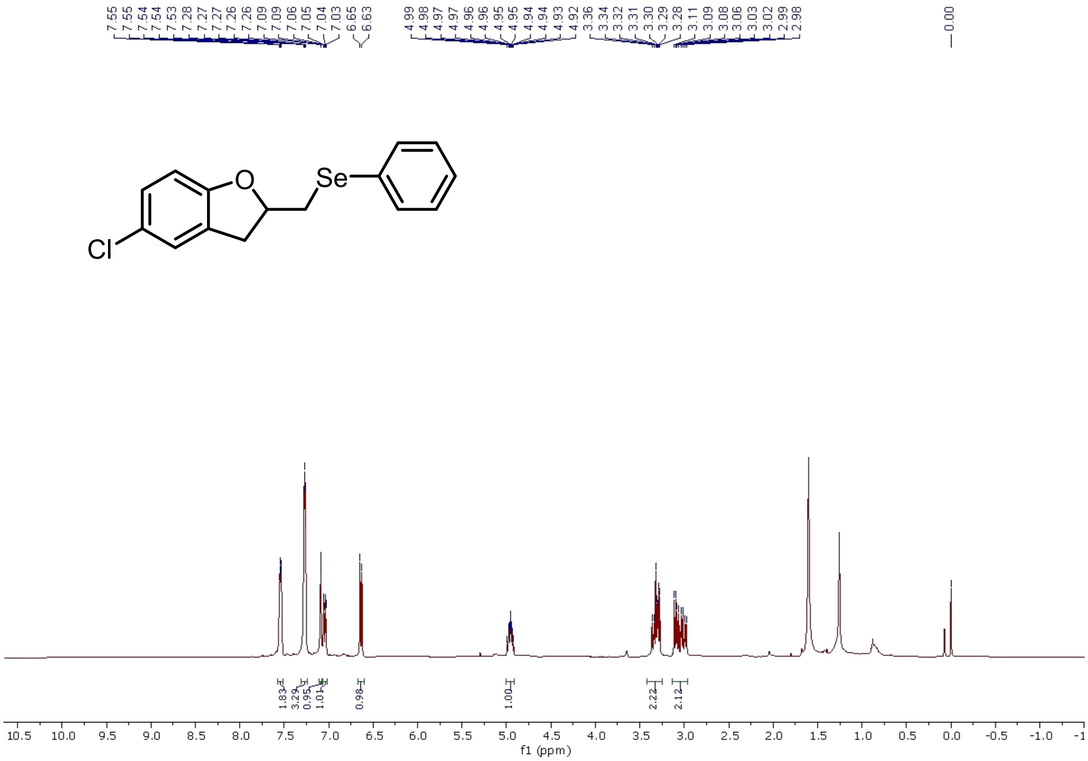

Figure S31. ^{1}H NMR (400 MHz, $CDCl_3$) spectrum of compound **3j**.

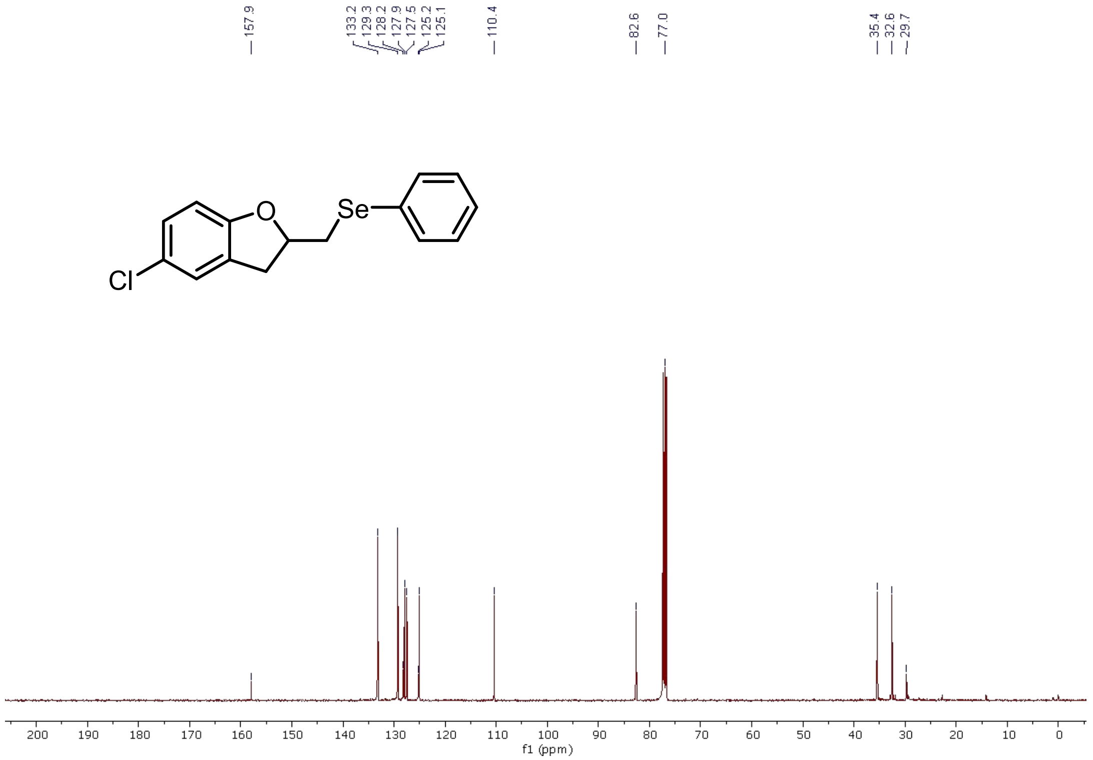

Figure S32. ^{13}C NMR (100 MHz, $CDCl_3$) spectrum of compound **3j**.

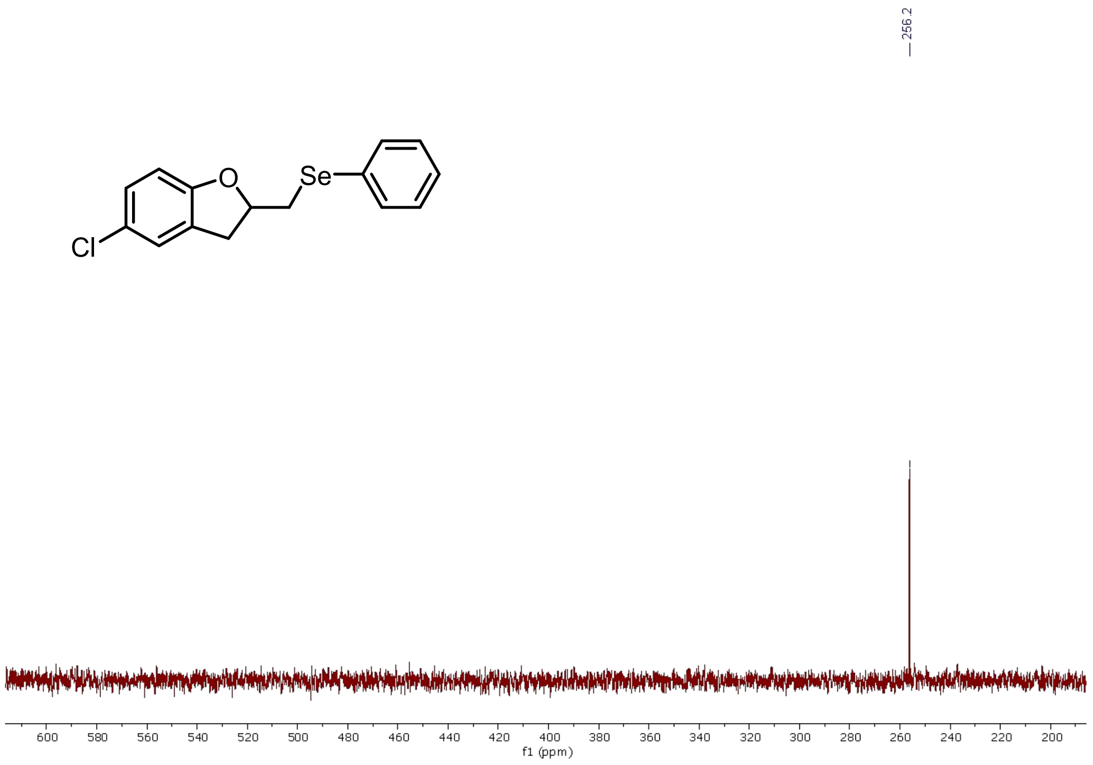

Figure S33. ^{77}Se NMR (76 MHz, $CDCl_3$) spectrum of compound **3j**.

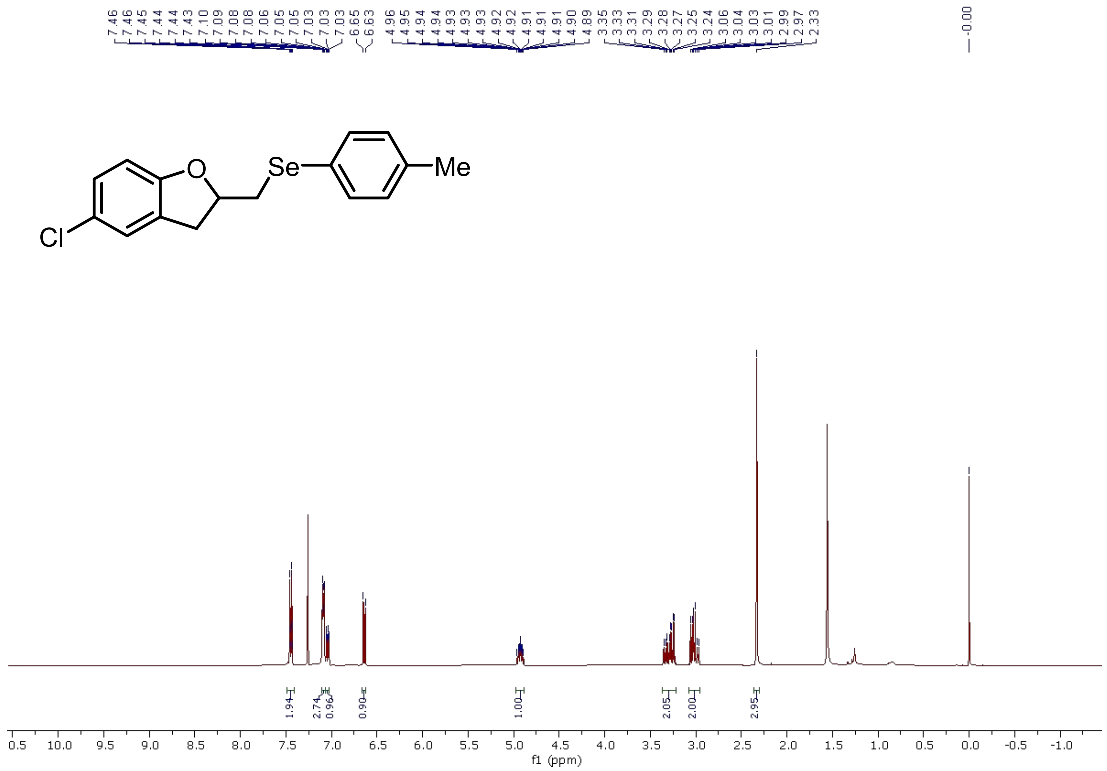

Figure S34. ^{1}H NMR (400 MHz, $CDCl_3$) spectrum of compound **3k**.

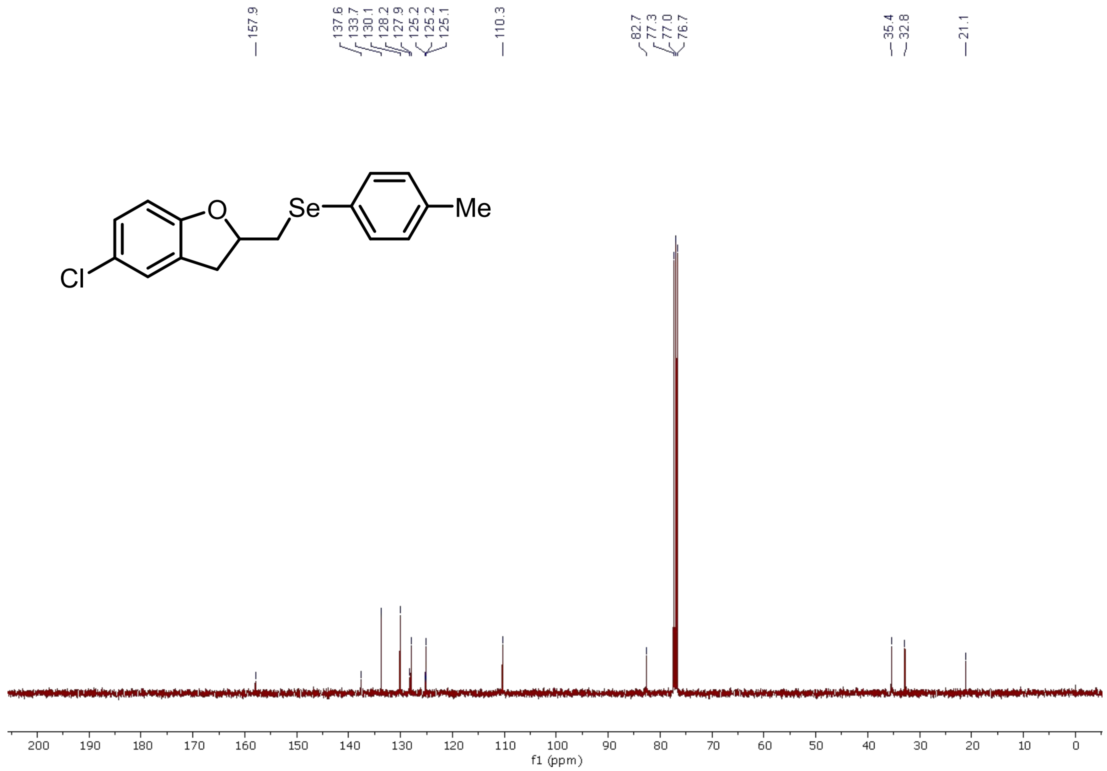

Figure S35. ^{13}C NMR (100 MHz, $CDCl_3$) spectrum of the compound **3k**.

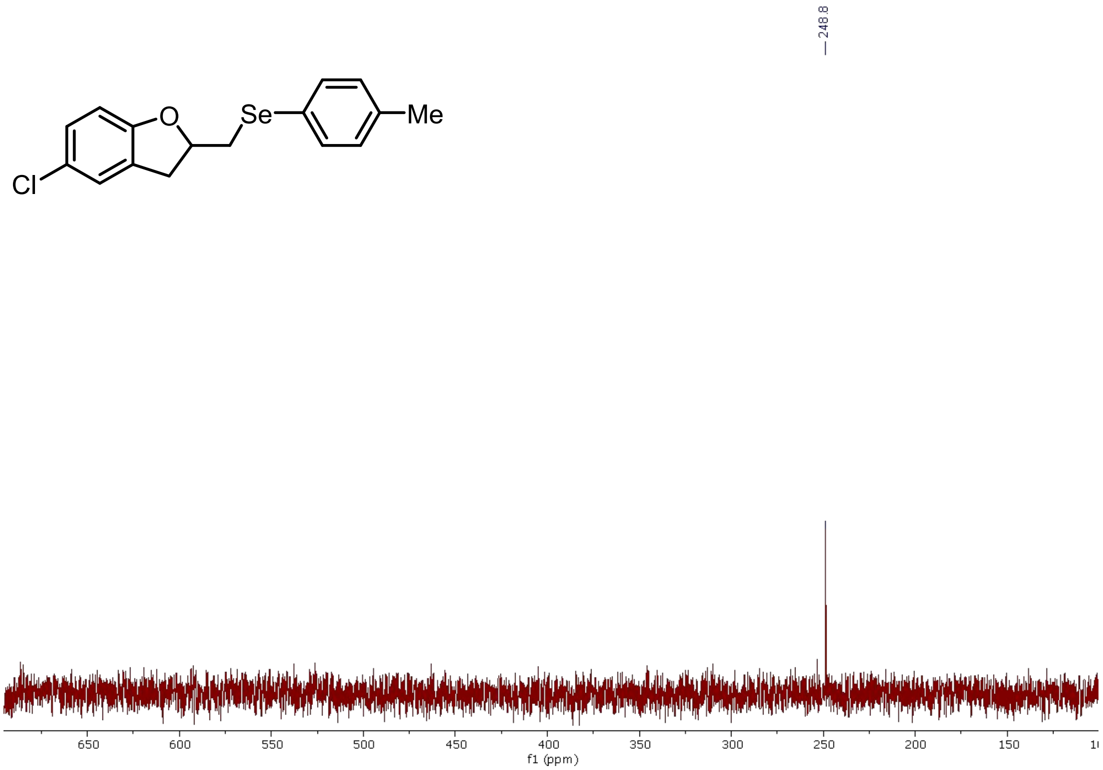

Figure S36. ^{77}Se NMR (100 MHz, $CDCl_3$) spectrum of compound **3k**.

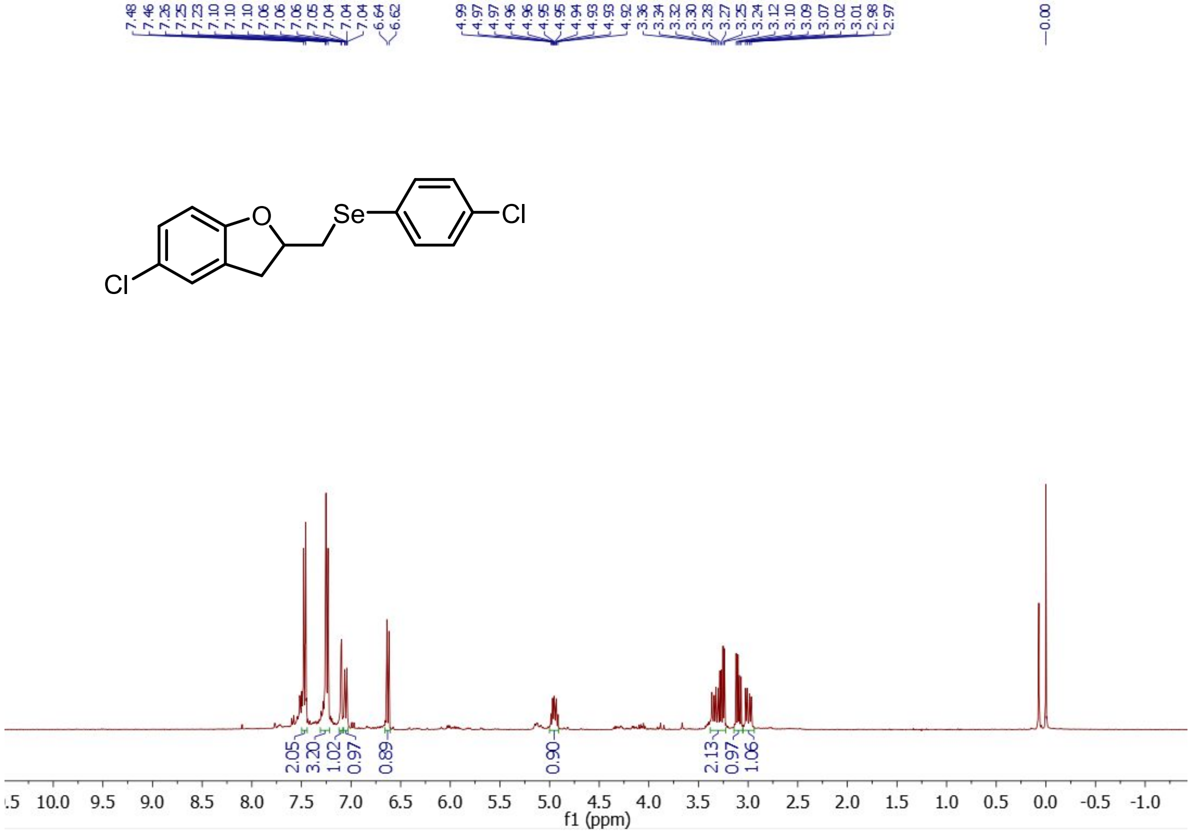

Figure S37. ^{1}H NMR (400 MHz, $CDCl_3$) spectrum of compound **3l**.

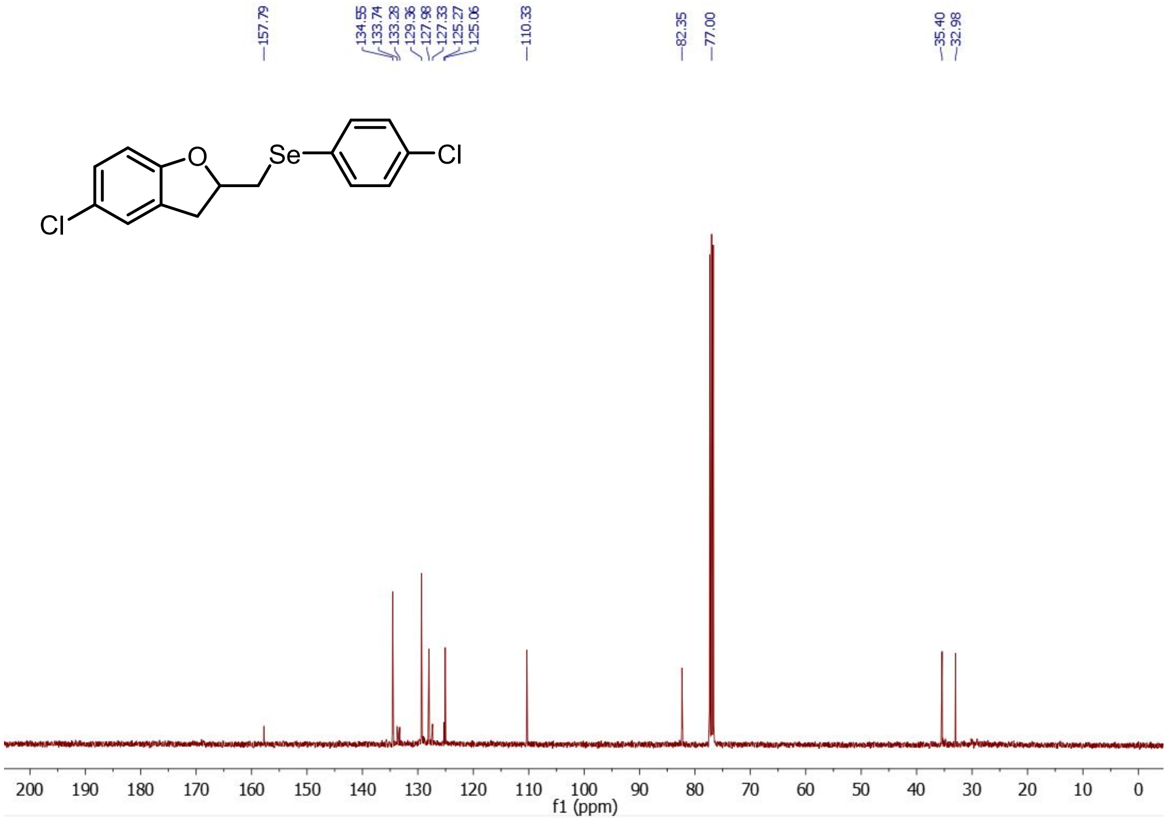

Figure S38. ^{13}C NMR (100 MHz, $CDCl_3$) spectrum of compound **3l**.

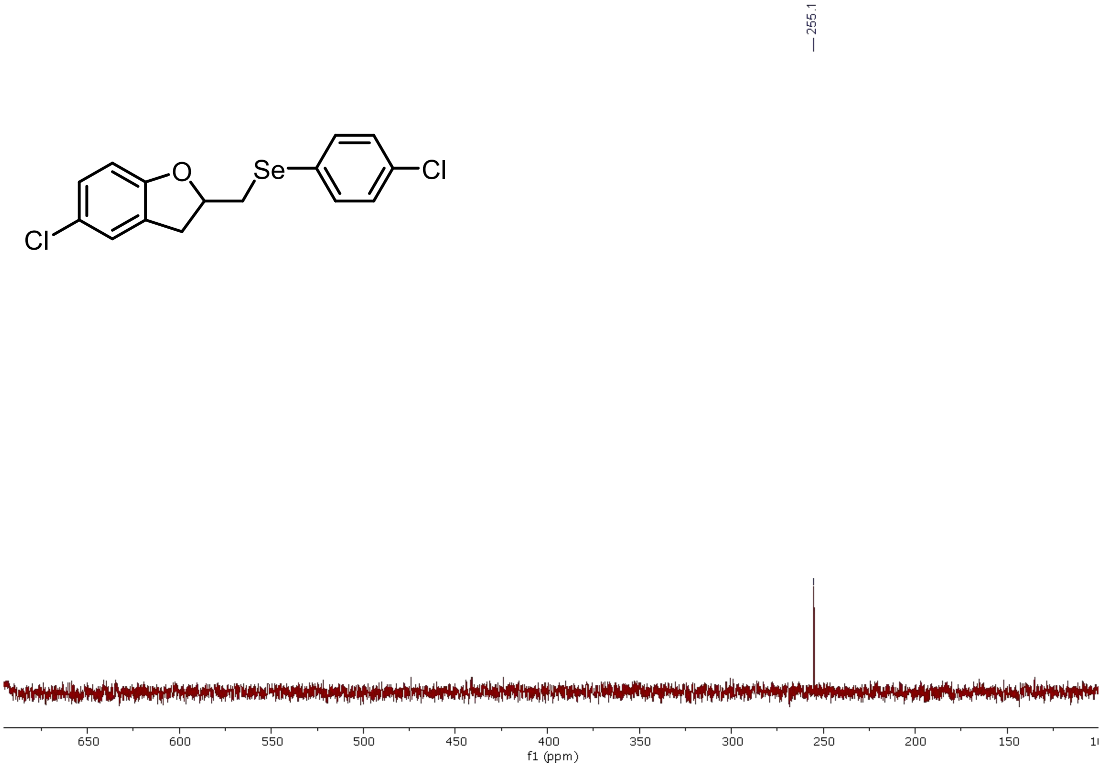

Figure S39. ^{77}Se NMR (100 MHz, $CDCl_3$) spectrum of compound **3l**.

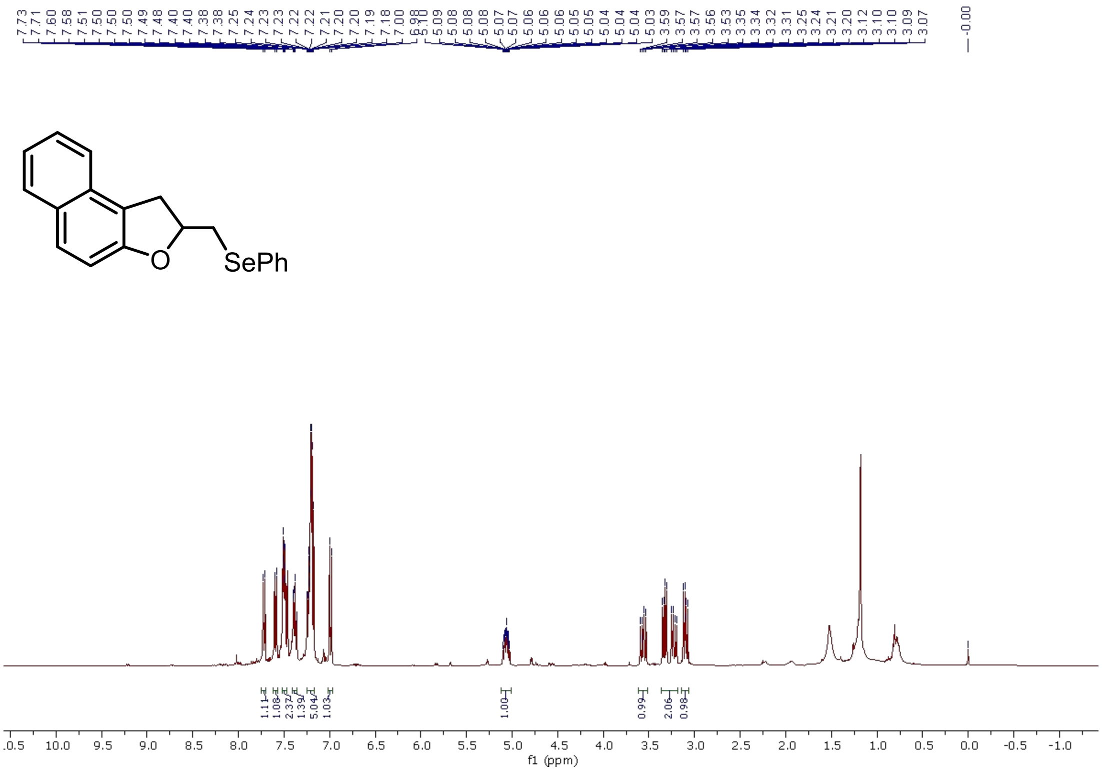

Figure S40. ^{1}H NMR (400 MHz, $CDCl_3$) spectrum of compound **3m**.

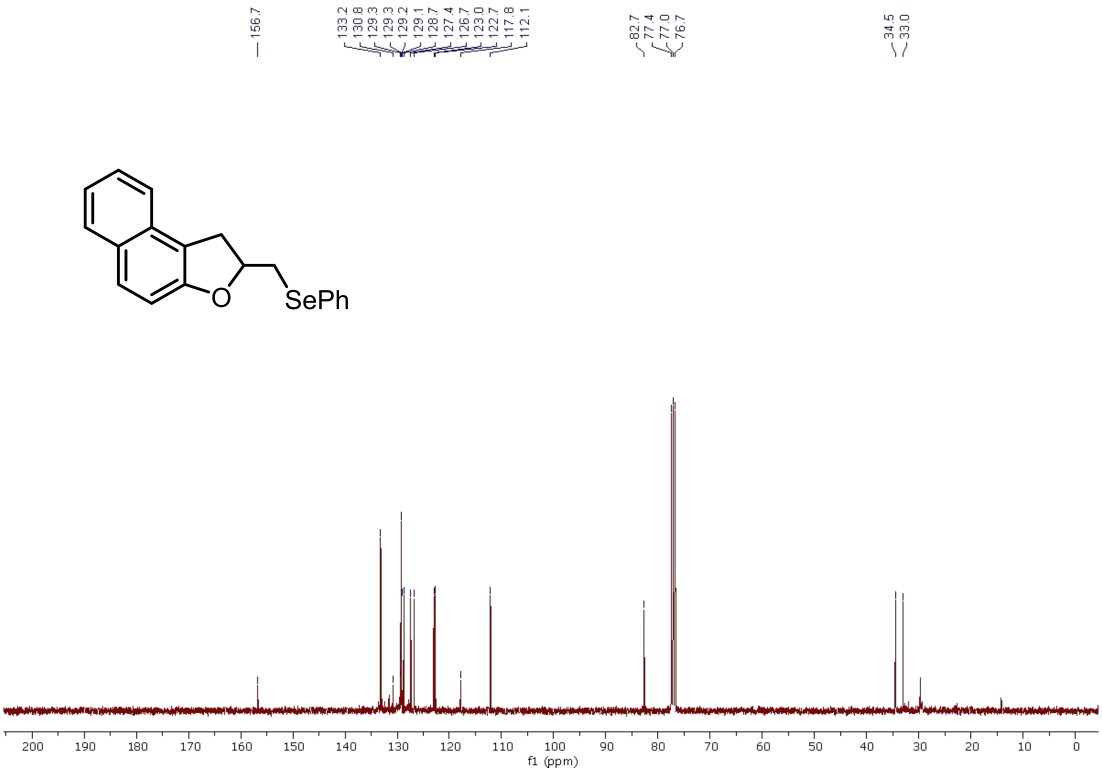

Figure S41. ^{13}C NMR (100 MHz, $CDCl_3$) spectrum of compound **3m**.

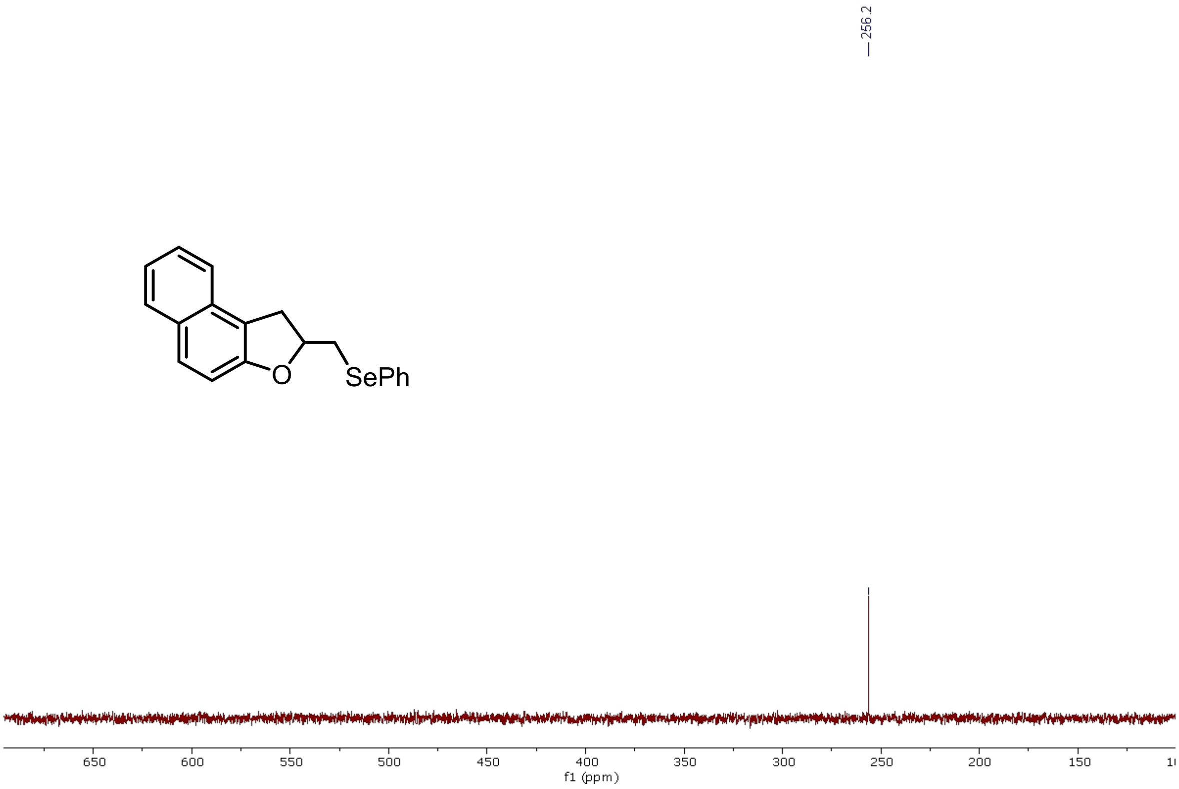

Figure S42. ^{77}Se NMR (100 MHz, $CDCl_3$) spectrum of compound **3m**.

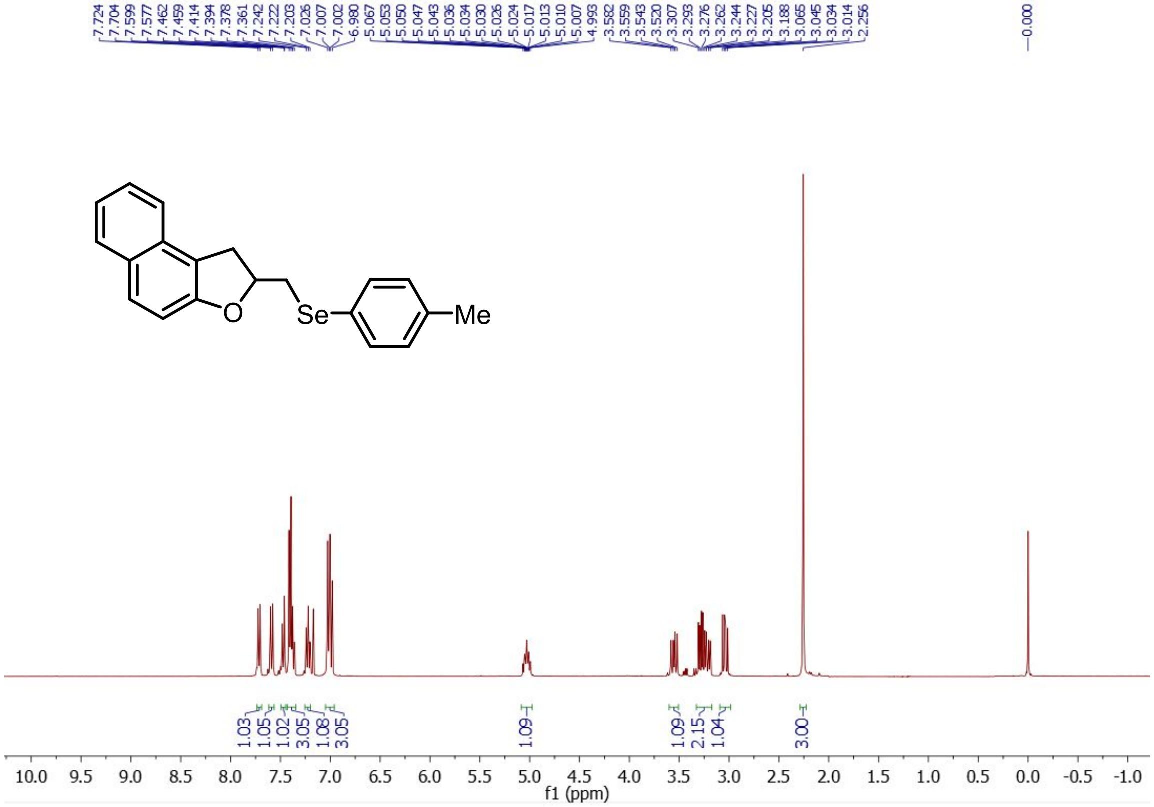

Figure S43. ^{1}H NMR (400 MHz, $CDCl_3$) spectrum of compound **3n**.

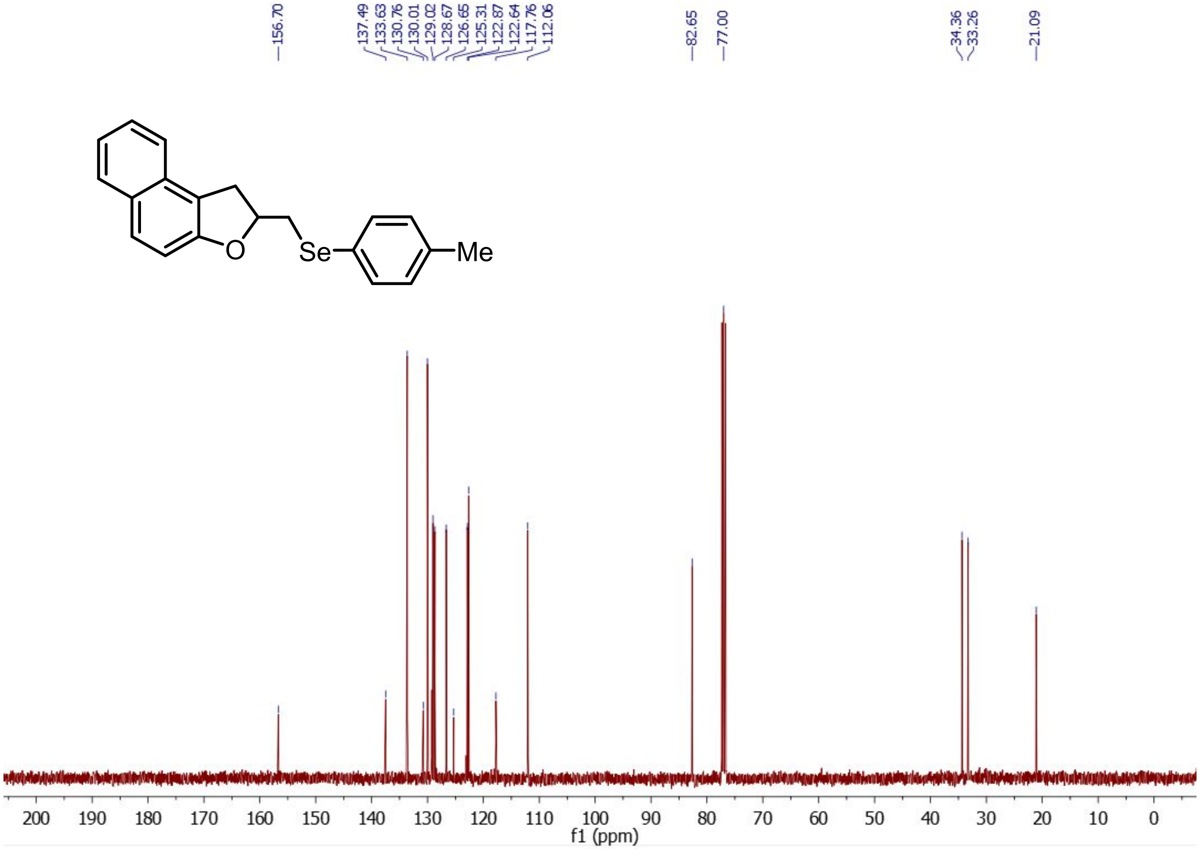

Figure S44. ^{13}C NMR (100 MHz, $CDCl_3$) spectrum of compound **3n**.

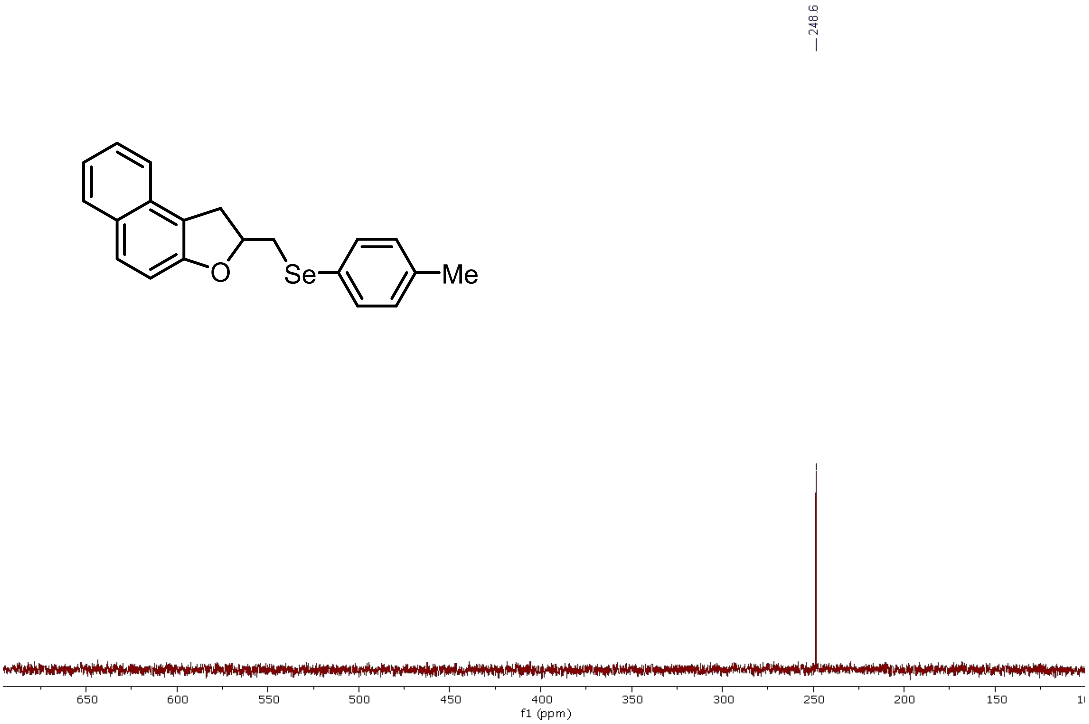

Figure S45. ^{77}Se NMR (100 MHz, $CDCl_3$) spectrum of compound **3n**.

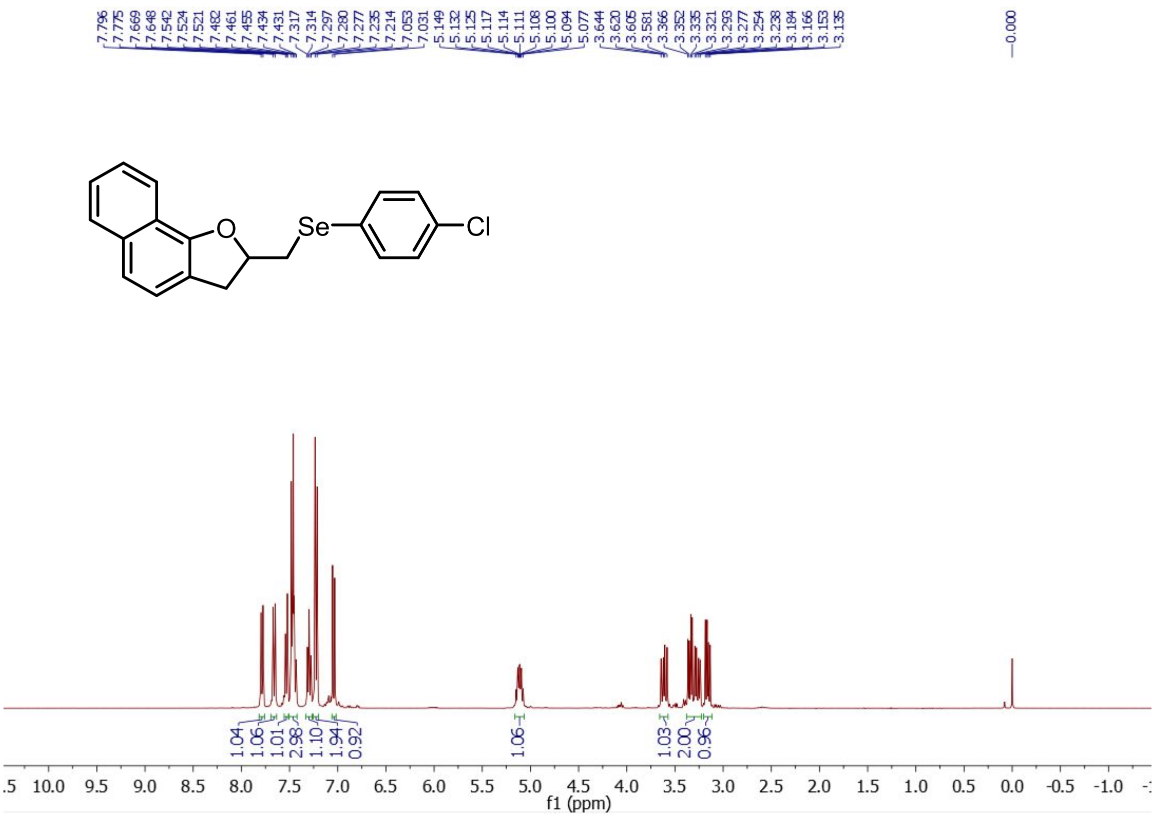

Figure S46. ^{1}H NMR (400 MHz, $CDCl_3$) spectrum of compound **3o**.

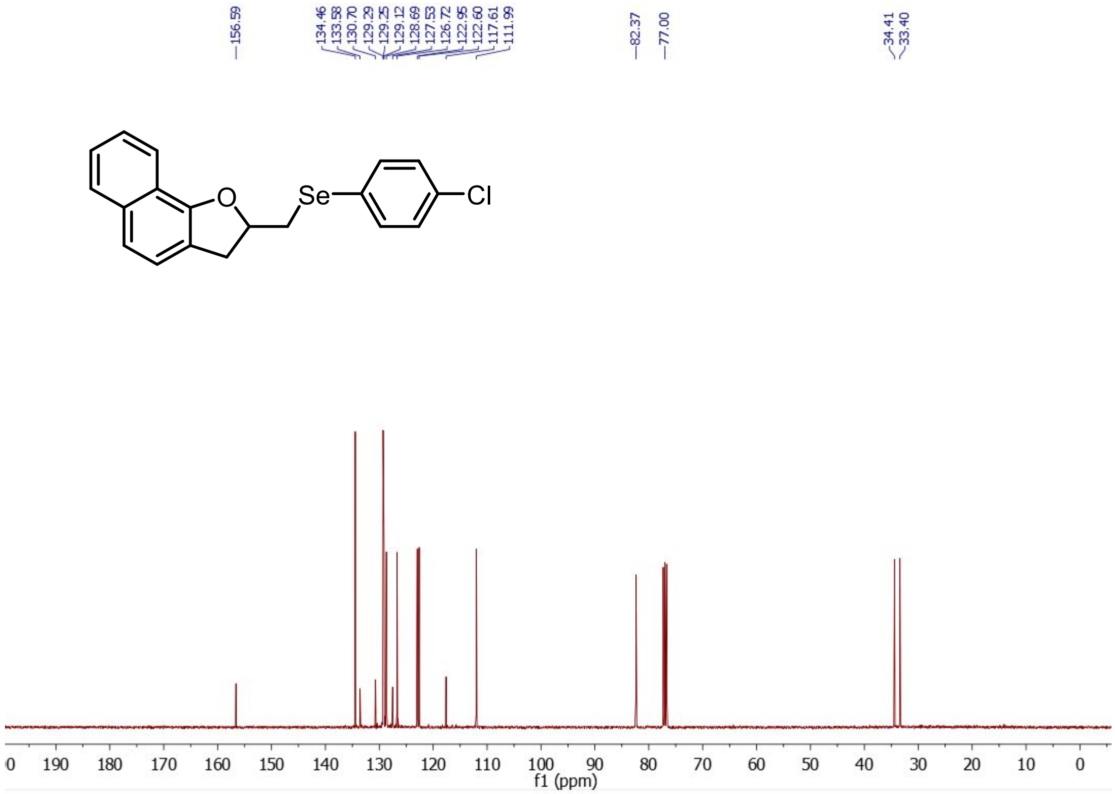

Figure S47. ^{13}C NMR (100 MHz, $CDCl_3$) spectrum of compound **3o**.

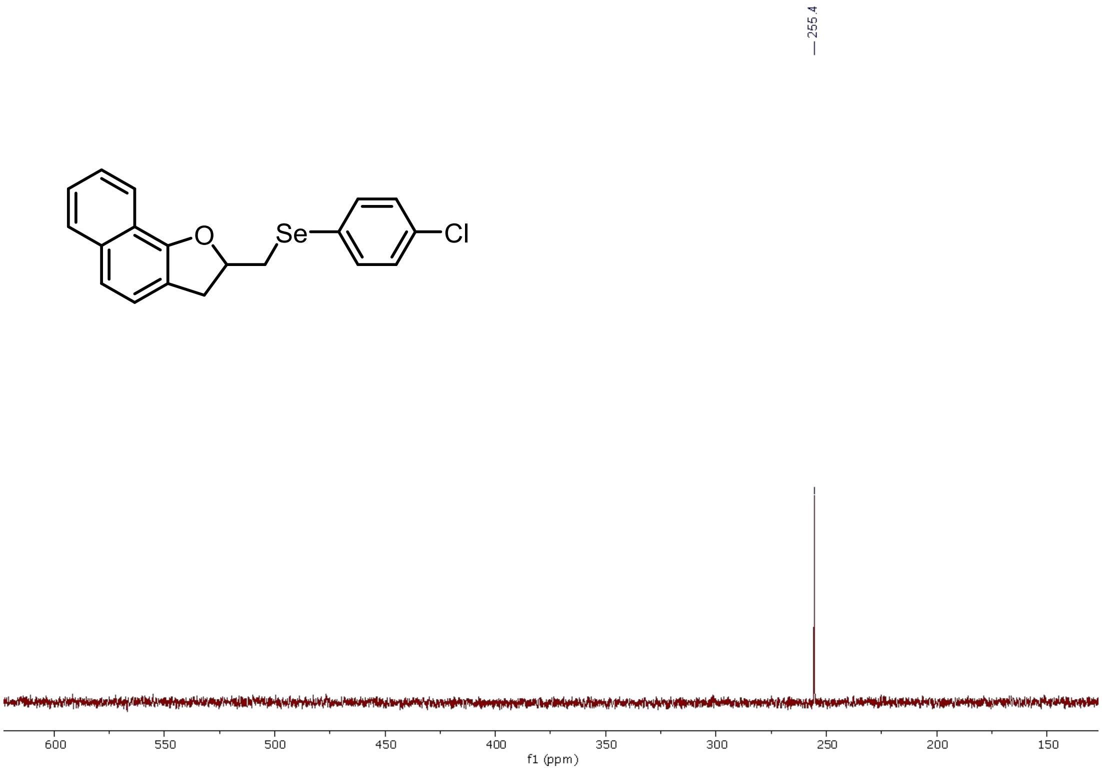

Figure S48. ^{77}Se NMR (100 MHz, $CDCl_3$) spectrum of compound **3o**.

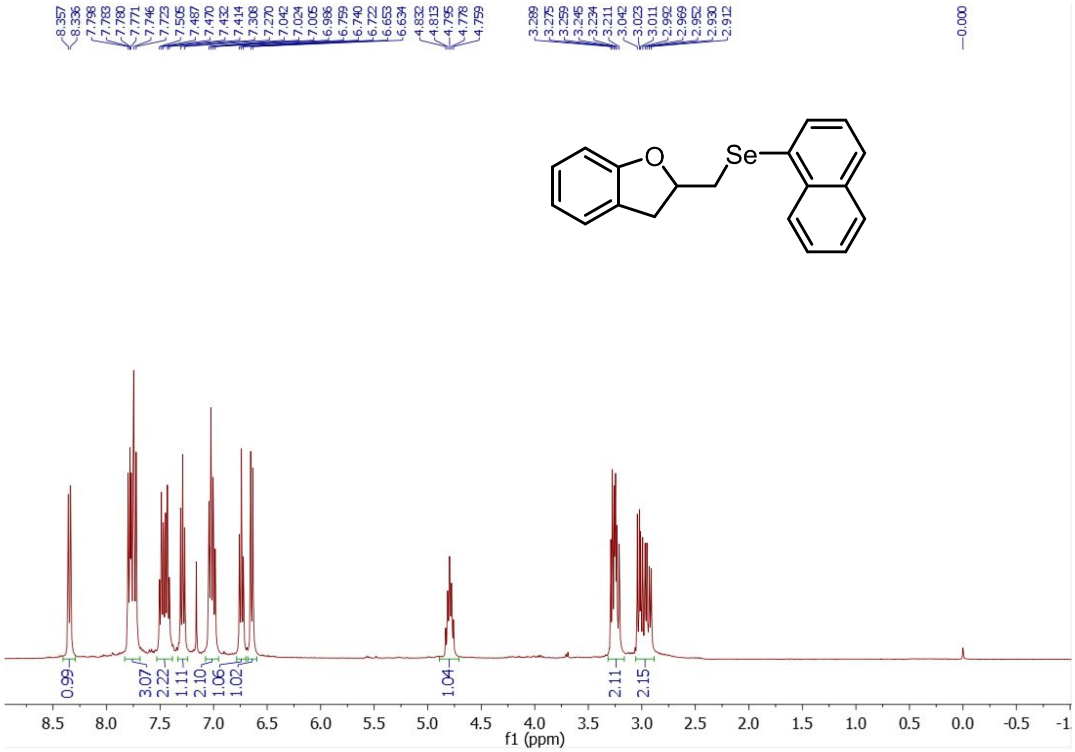

Figure S49. ^{1}H NMR (400 MHz, $CDCl_3$) spectrum of compound **3p**.

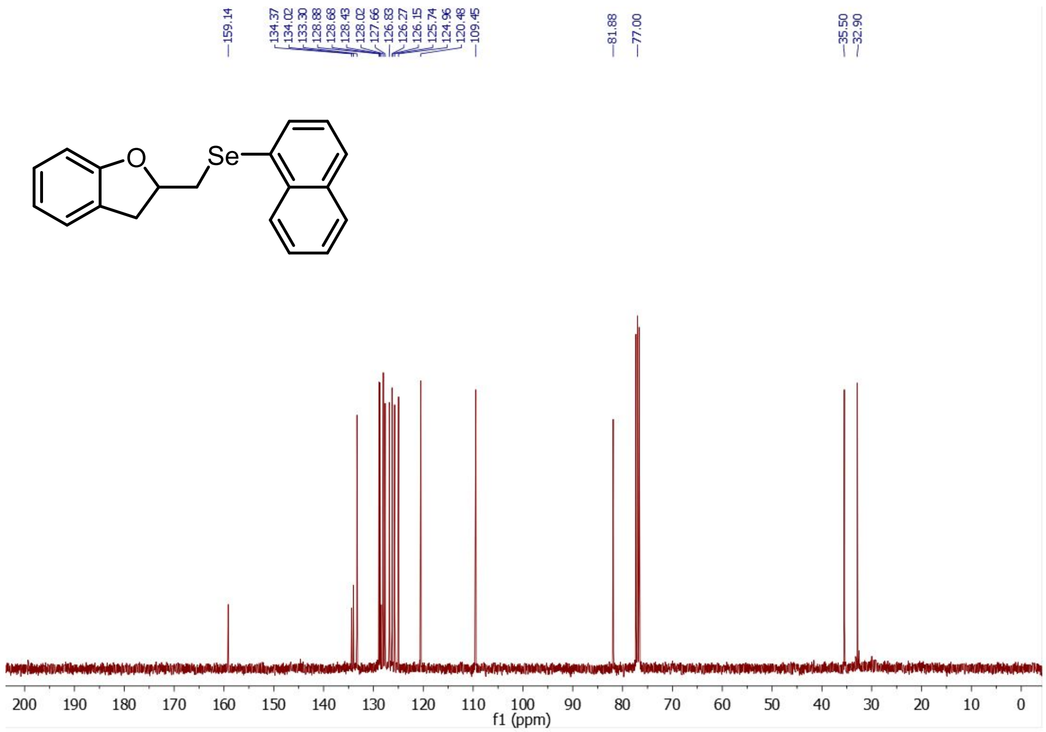

Figure S50. ^{13}C NMR (100 MHz, $CDCl_3$) spectrum of compound **3p**.

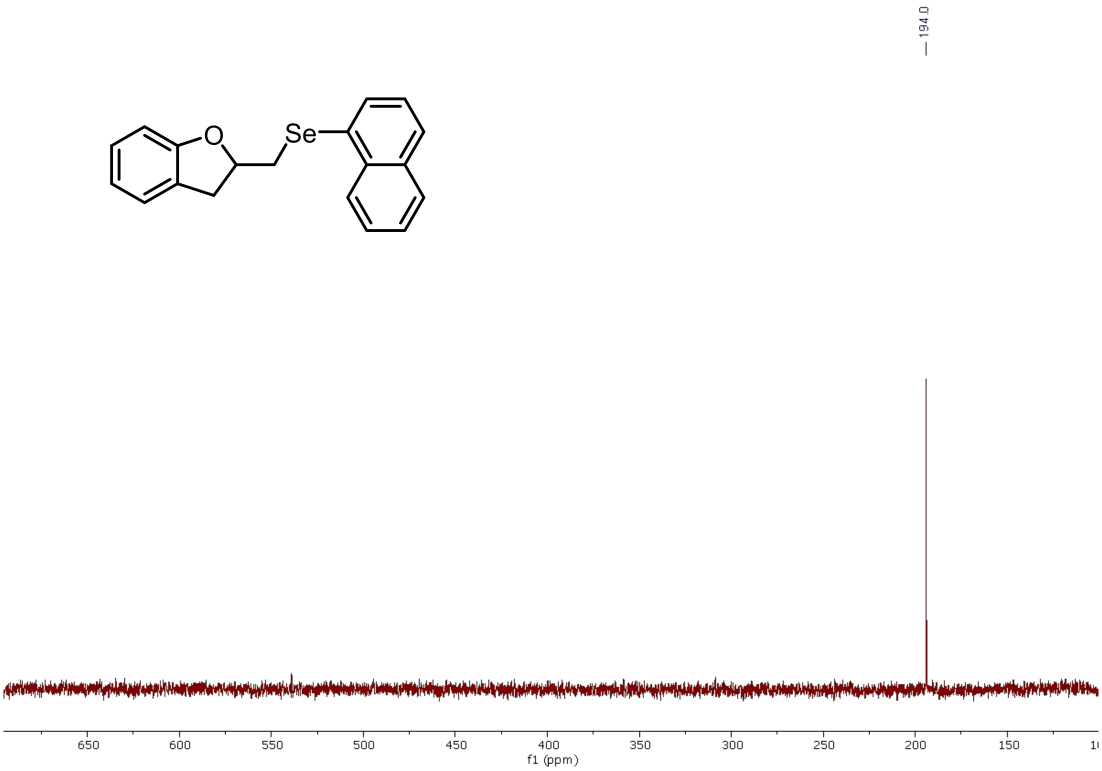

Figure S51. ^{77}Se NMR (100 MHz, $CDCl_3$) spectrum of compound **3p**.

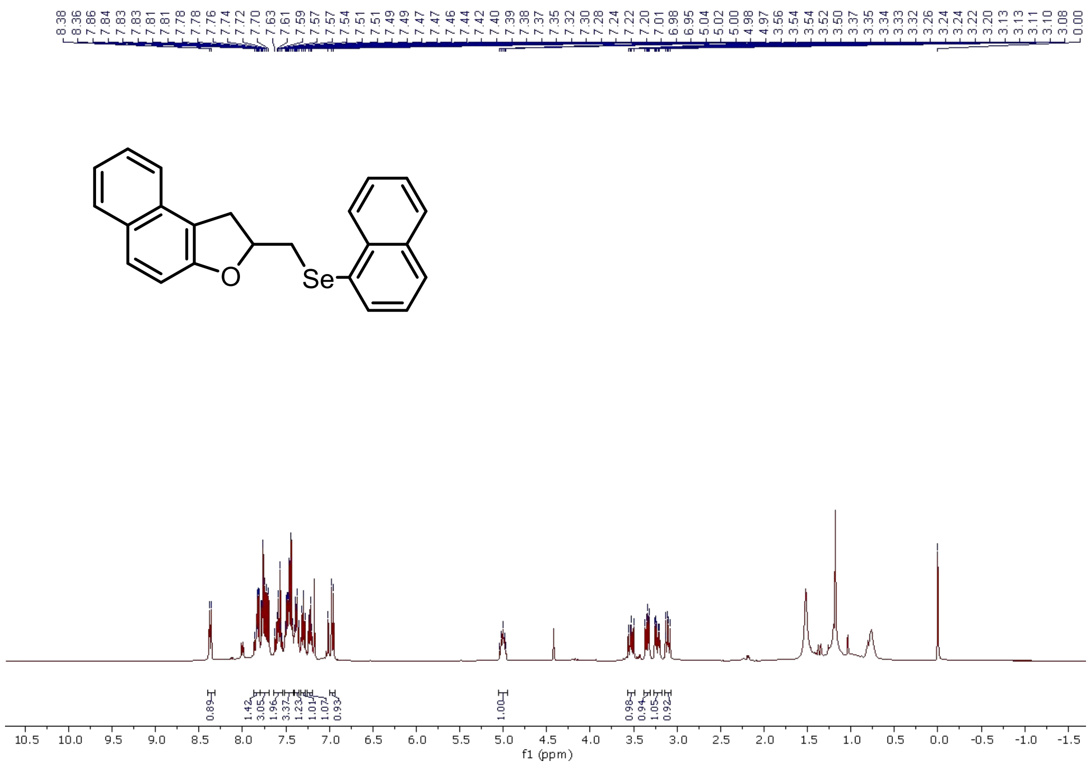

Figure S52. ^{1}H NMR (400 MHz, $CDCl_3$) spectrum of compound **3q**.

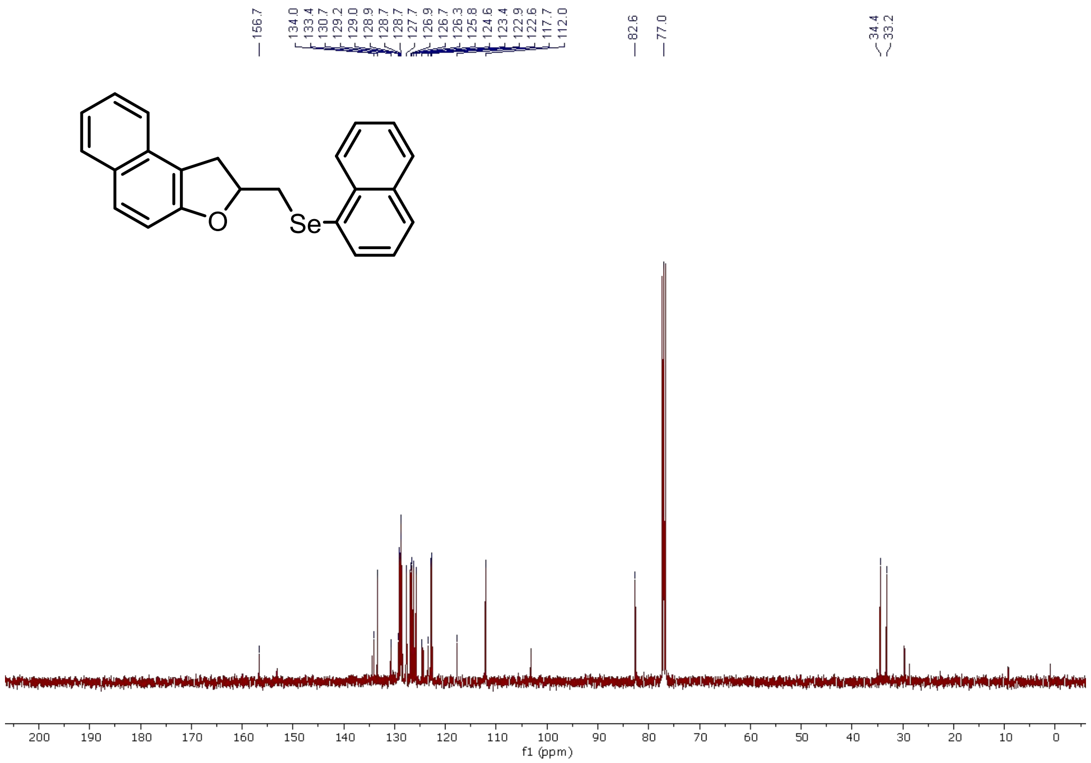

Figure S53. ^{13}C NMR (100 MHz, $CDCl_3$) spectrum of compound **3q**.

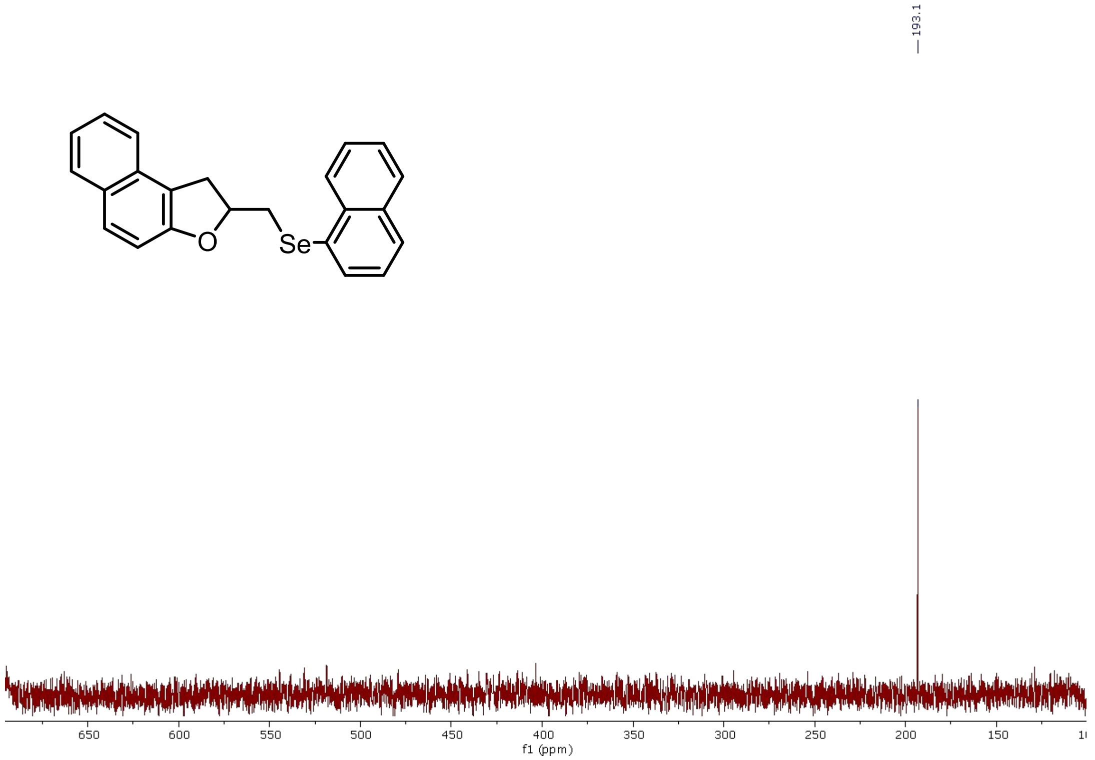

Figure S54. ^{77}Se NMR (76 MHz, $CDCl_3$) spectrum of compound **3q**.

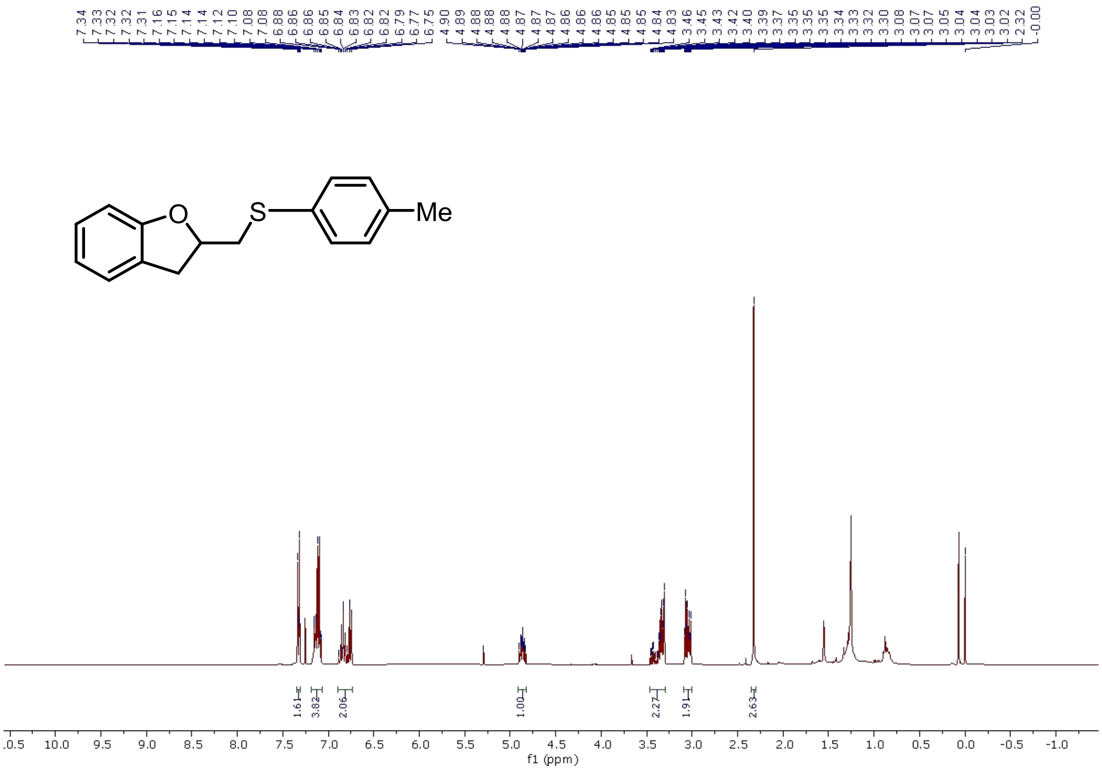

Figure S52. ^{1}H NMR (400 MHz, $CDCl_3$) spectrum of compound **3r**.

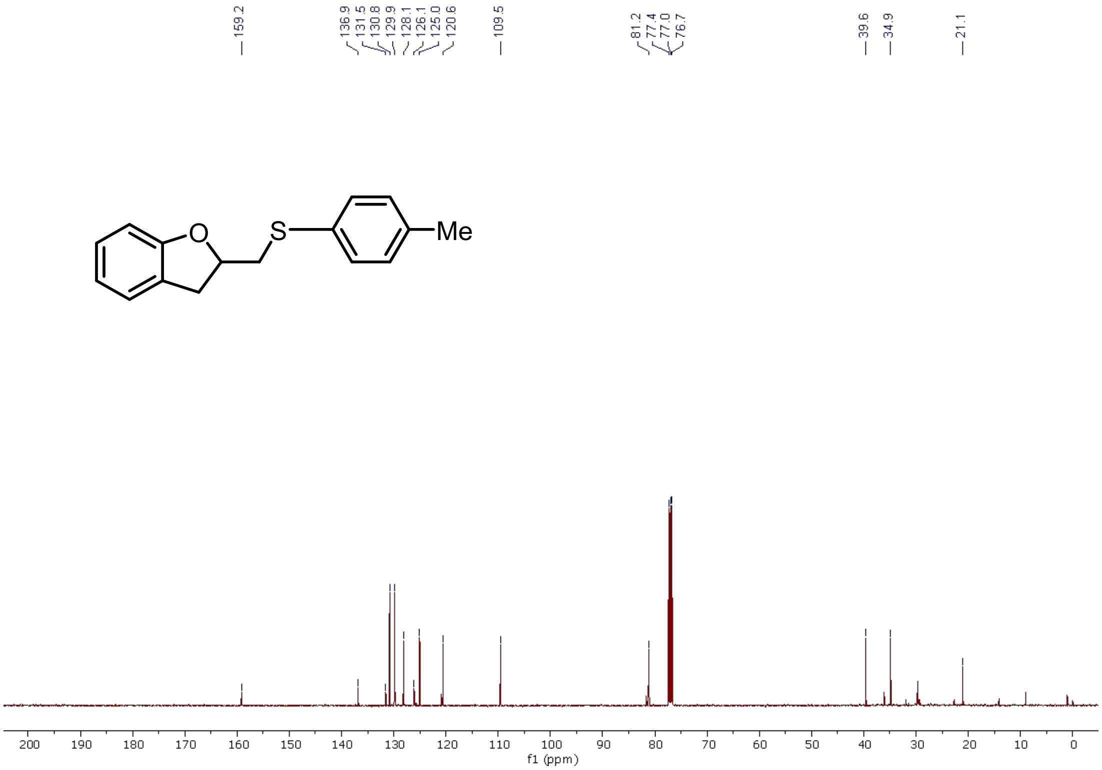

Figure S53. ^{13}C NMR (100 MHz, $CDCl_3$) spectrum of compound **3r**.
